# Identification of an optimal method for extracting RNA from human skin biopsy, using domestic pig as a model system

**DOI:** 10.1038/s41598-019-56579-5

**Published:** 2019-12-27

**Authors:** Ene Reimann, Kristi Abram, Sulev Kõks, Külli Kingo, Alireza Fazeli

**Affiliations:** 1 Department of Pathophysiology, University of Tartu, Tartu, Estonia; 2 Department of Dermatology, University of Tartu, Tartu, Estonia; 3 Clinic of Dermatology, Tartu University Hospital, Tartu, Estonia; 4 Centre for Comparative Genomics, Murdoch University, Perth, 6150 Australia; 5 The Perron Institute for Neurological and Translational Science, Perth, 6009 Australia; 6 Academic Unit of Reproductive and Developmental Medicine, Department of Oncology and Metabolism, The Medical School, University of Sheffield, Sheffield, United Kingdom

**Keywords:** Gene expression analysis, Transcriptomics, Genetics research

## Abstract

To evaluate skin tissue gene expression patterns correctly, extracting sufficient quantities of good quality RNA is essential. However, RNA extraction from skin tissue is challenging, as the hyaluronic acid-collagen matrix is extremely difficult to homogenize. Although there are multiple ways to extract RNA from skin, there are no comparative studies that identify the most critical steps, e.g. sample collection, storage and homogenization. We analysed the various steps involved in RNA extraction (i.e. biopsy collection as dry biopsy or into nucleotide stabilizing reagents, different storage conditions, enzymatic digestion, stator-rotor and bead motion-based homogenizing combined with column-based RNA purification). We hypothesised that domestic pig skin is applicable as a model for human skin studies. Altogether twenty different workflows were tested on pig skin and the four most promising workflows were tested on human skin samples. The optimal strategy for extracting human skin RNA was to collect, store and homogenize the sample in RLT lysis buffer from the RNeasy Fibrous Tissue Kit combined with beta-mercaptoethanol. Both stator-rotor and bead motion-based homogenizing were found to result in high quality and quantity of extracted RNA. Our results confirmed that domestic pig skin can be successfully used as a model for human skin RNA studies.

## Introduction

Skin is a challenging tissue for gene expression analysis. Firstly, because it is exposed to the external environment it is contaminated with various biomolecules including nucleases. Furthermore, due to high stability of hyaluronic acid-collagen matrix, skin is one of the most difficult tissues to homogenize^1^. The present study aimed to find an optimal solution for overcoming the difficulties in extracting RNA from human skin tissue by testing the relative benefits and disadvantages of several different but widely used methods.

There are several methods available to extract RNA from skin. However, there have been no comparative and systematic investigations aimed at evaluating sample collection, storage and homogenizing steps. These are the most critical steps when working with skin tissue. Currently, two widely used strategies to block the activity of RNases are either immediate freezing after sample collection or applying stabilizing reagents at room temperature and/or lower temperatures^2–5^. Homogenization of skin tissue is usually achieved via mincing with razor blades, using mechanical homogenizers or using a mortar and pestle in combination with liquid nitrogen, which is an efficient, but time-consuming process^6–10^. Additionally, enzymatic digestion of hyaluronic acid-collagen matrix with collagenase and hyaluronidase prior to mechanical homogenization might increase the efficiency of homogenization (sisweb.com, 05/2009)), however, there is no clear evidence about its efficiency. Two commonly used solutions for homogenization contain phenol and guanidine thiocyamte (QIAzol Handbook, 01/2009), (TRIzol Reagent, 2016), which commercial lysis buffers often combine with beta-mercaptoethanol (BME) (Qiagen RLT buffer, 2018), and which have both lysis and RNA inhibiting capacities.

A critical limitation for accurate and meaningful comparisons of different RNA extraction methods for human tissue is the scarcity and difficulty of obtaining sufficient tissue itself. Thus, in the current investigation we initially used domestic pig skin tissue for comparing different techniques. Pig skin is widely used in various fields of dermatological research, as it is easily accessible and has similar skin structure to humans (i.e.– spare hair coat, similar general thickness of skin layers, thick epidermis with well differentiated understructure, a dermis with well-differentiated papillary body, a large content of elastic tissue, and similar architecture of collagen fibres). These characteristics outweigh the dissimilarities, such as higher vascularisation in human and higher fat content in pig skin^11–15^. Hence, domestic pig has successfully been used in studies regarding percutaneous absorption^11^, different skin injuries such as bite marks^16^, burns and autografting^17^, diseases such as melanoma^18^, and in toxicology studies^19^.

The present study aimed to find an optimal solution for overcoming the difficulties in extracting RNA from human skin tissue by testing the relative benefits and disadvantages of different but widely used methods. We tested (1) four different sample collection/storing strategies (dry biopsy, different stabilising solutions at diverse temperatures), (2) the presence or absence of enzymatic treatment prior homogenizing, (3) two different homogenizing solutions (phenol-based and BME-based), and (4) two different homogenizing instruments (stator-rotor and bead (ceramic and metal) motion-based homogenizing). The final RNA purification step combined with DNase treatment was conducted on silica-membrane columns. Altogether twenty different workflows (WFs) were analysed (WF names and strategies each of these involve are presented in Table 1). Additionally, we analysed whether domestic pig is applicable as a model system for human skin studies regarding RNA extraction and which extraction strategy results in RNA with the highest quality and quantity.

**Table 1:** Description of different workflows (WF).

Workflow name	Sample collection	Enzymatic digestion	Homogenizing buffer	Homogenizing instrument and tube	RNA extraction and DNase treatment
WF1_D	APTR	Yes	QIAzol	Fastprep: D	RNeasy Fibrous Tissue Mini kit + DNase I
WF1_S	APTR	Yes	QIAzol	Fastprep: S	
WF2_D	APTR	No	QIAzol	Fastprep: D	
WF2_S	APTR	No	QIAzol	Fastprep: S	
WF3_D*	QIAzol	No	QIAzol	Fastprep: D	
WF3_S	QIAzol	No	QIAzol	Fastprep: S	
WF4_D	APTR	Yes	BME + LB	Fastprep: D	
WF4_S	APTR	Yes	BME + LB	Fastprep: S	
WF5_D	Dry biopsy	Yes	BME + LB	Fastprep: D	
WF6_D	APTR	No	BME + LB	Fastprep: D	
WF6_S	APTR	No	BME + LB	Fastprep: S	
WF7_D*	BME + LB	No	BME + LB	Fastprep: D	
WF7_S	BME + LB	No	BME + LB	Fastprep: S	
WF8_D	Dry biopsy	No	BME + LB	Fastprep: D	
WF9_M	APTR	Yes	QIAzol	GentleMACS: M	
WF10_M	APTR	No	QIAzol	GentleMACS: M	
WF11_M*	QIAzol	No	QIAzol	GentleMACS: M	
WF12_M	APTR	Yes	BME + LB	GentleMACS: M	
WF13_M	APTR	No	BME + LB	GentleMACS: M	
WF14_M*	BME + LB	No	BME + LB	GentleMACS: M	

QIAzol - QIAzol Lysis reagent, APTR - Allprotect Tissue Reagent, BME + LB - RTL lysis buffer from RNeasy Fibrous Tissue Mini kit containing beta-mercaptoethanol, Fastprep D/S - Fastprep-24 instrument with lysing matrix D or S tubes, GentleMACS M - GentleMACS Dissociator with M tubes. The D, S or M in WF name refer to the homogenizing tube (Fastprep lysing matrix D or S tubes or GentleMACS M tubes, respectively) applied in this WF.

*These workflows were used for validation with human samples.

It is important to note that our emphasis in this study was to see if a phenol-based solution increases the homogenizing efficiency and RNA quality compared to applying commercial lysis buffer containing BME. Thus, during this study we did not compare different phenol-based solutions (such as TRIzol Reagent (TRIzol Reagent, 2016) and QIAzol Lysis Reagent (QIAzol Handbook, 01/2009)) with each other. Additionally, we did not test an RNA extraction protocol with phenol-based solution alone, but only as combined with silica-membrane columns. The latter was due to our previous experience that without additional on-columns purification, the RNA purity values tend to be poor (unpublished observations by Ene Reimann).

## Results

### Correlations between the weight of skin biopsy (input) and the amount of total RNA (output)

For domestic pig, the weight range of collected biopsies prior to RNA extraction was 14 to 76 mg (average 40.1 mg ± SD 17.56) (Table 1S in Supplementary MaterialsSupplementary Materials). The average tissue weights of samples from each pig separately were: Pig 1 = 55.0 mg ± SD 15.8, Pig 2 = 34.2 mg ± SD 12.1, Pig 3 = 31.1 mg ± SD 14.4. The total RNA amount was in a range between 0.43 to 52.0 μg (average 9.9 μg ± SD 10.1). The average RNA yields of samples from each pig separately were: Pig 1 = 10.8 μg ± SD 8.9, Pig 2 = 12.4 μg ± SD 13.7, Pig 3 = 6.5 μg ± SD 5.3. When analysing the pooled data from pig samples, neither the input tissue weights nor the output RNA amounts passed the normality test (D’Agostino and Pearson omnibus normality test; n = 60, P_tissueweight_ < 0.013 and P_RNAoutput_ < 0.0001). We found a significant association between the tissue input and RNA output: 27% of the variability in RNA output was explained by input tissue amount (R = 0.52, P < 0.0001, Fig. 2SA in Supplementary Materials). If the data were analysed separately for each pig, the weight range passed the normality test, but the total RNA amounts did not, and thus a Spearman’s correlation was applied (Pig 1: n = 20, P_tissueweight_ < 0.3552, P_RNAoutput_ < 0.0009; Pig 2: n = 20, P_tissueweight_ < 0.3348, P_RNAoutput_ < 0.0021; Pig 3: n = 20, P_tissueweight_ < 0.1466, P_RNAoutput_ < 0.0042). We found that only samples from one pig showed a significant correlation between tissue input and RNA output (Pig 1: R = 0.62, P = 0.0038; Pig 2: R = 0.44, P = 0.0502, Pig 3: R = 0.37, P = 0.1124).

For human samples, the weight range of biopsies was 24 to 134 mg (average 64.67 mg with ±SD 33.01). After RNA extraction, the total RNA amount was in a range of 2.3 to 13.3 (μg) (average 5.8 μg with ±SD 3.0). If applying the pooled values from human skin biopsies, the weight range passed the normality test, but the total RNA amount did not (n = 12, P_tissueweight_ < 0.2658, P_RNAoutput_ < 0.009) and according to the Spearman’s correlation analysis there was no correlation between the weight of skin biopsy and extracted total RNA amount (correlation coefficient was 0.46 and P = 0.13; Fig. 2SB in Supplementary Materials). For human samples, we were unable to perform separate analyses for each individual, as there were only two samples per individual.

### Comparison of different sample collection and storage strategies

For pig skin samples, four sample collection/storing strategies were tested: sample collection as dry biopsies with immediate freezing, sample collection into Allprotect Tissue Reagent (APTR), collection into QIAzol Lysis reagent (QIAzol) or collection into RLT lysis buffer containing additional beta-mercaptoethanol (BME + LB) with delayed freezing. Samples collected as dry biopsy or into BME + LB resulted in higher total RNA amounts and quality values (optical density (OD) rates, RNA Integrity Number (RIN)) (Fig. 3S in Supplementary Materials). All four collection strategies resulted in RNA with high RIN values (≥8.5), however, the APTR and QIAzol methods tended to result in lower OD 260/230 values (dry biopsy and BME + LB values were 1.8 and 1.9, respectively; APTR and QIAzol values were 1.6 and 1.7, respectively).

### The effect of enzymatic digestion on homogenizing efficiency

The hyaluronidase-collagenase digestion was tested for domestic pig samples collected as dry biopsy or into APTR (WF1, WF4, WF5, WF9, WF12). Fifty percent of enzymatically digested tissues (WF9 and WF12) and 33.3% of non-digested biopsies (WF10 and WF13) homogenized with GentleMACS Dissociator with M tubes were fully homogenized, however, the difference was not statistically significant. In case of the Fastprep-24 instrument with lysing matrix D or S tubes, all enzymatically treated skin samples remained visually intact. Furthermore, after incubation for 2 hours at 37 °C, the RNA quality decreased rapidly (P < 0.0001); the average RINs with and without enzymatic digestion were 2.4 (±SD 1.6) and 8.8 (±SD 0.6), respectively.

### Comparison of homogenizing buffers

The next step in the RNA extraction workflows after collection/storage and/or enzymatic digestion was the mechanical homogenization, for which QIAzol and BME + LB were tested as homogenizing buffers. Although we found the homogenizing efficiency to be similar between these two solutions, skin samples homogenized in BME + LB resulted in higher total RNA amounts, RNA purity values (OD A260/280 and A260/230 values) and RIN numbers compared to samples homogenized in QIAzol (Fig. 4S in Supplementary Materials). However, the amount of DNA contamination was higher for BME + LB samples.

### Comparison of rotor and stator and bead motion-based homogenizers

Two different homogenizing methods were compared in the present study: tissue treatment with bead beating (Fastprep-24 Instrument) and tissue fast spinning in tubes with stator and rotor (GentleMACS Dissociator). The Fastprep Instrument with D tubes (ceramic beads) or S tubes (metal beads) was not able to fully homogenize the skin samples – after five cycles, the homogenizing solution was cloudy, but the tissue piece remained intact. Conversely, the GentleMACS Dissociator with M tubes was able to fully homogenize 8 samples out of 18 (44.4%) after two cycles; the rest of the samples had similar results to the Fastprep Instrument. Additionally, comparing these two instruments and three tubes, the achieved RNA quantity and quality values were similar (Fig. 5S in Supplementary Materials).

### Most successful workflows for extracting RNA from domestic pig skin samples were applied to human skin biopsies

**Figure 1:**
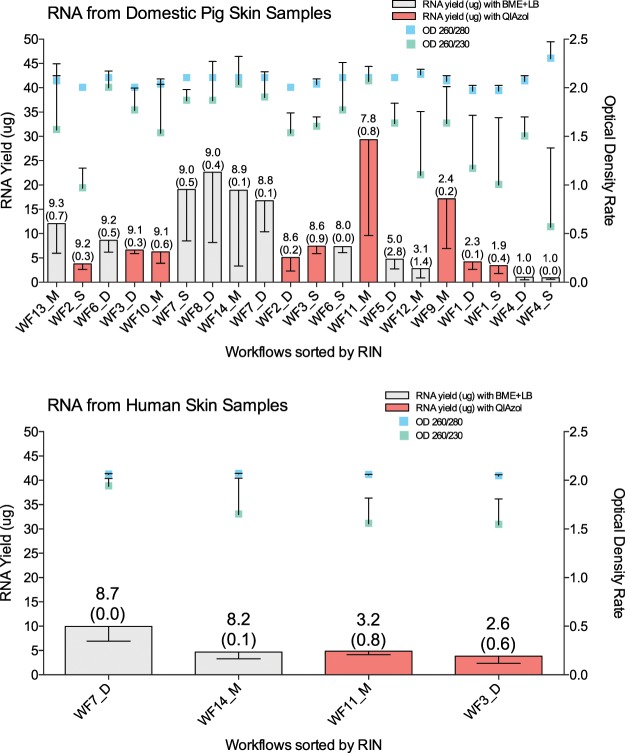
RNA quality and quantity values received by applying different workflows ordered according to the RIN values (starting from the workflow with highest RIN value). The RIN values are represented on top of the columns together with ±SD in brackets. The error bars represent the ±SD. (**A**) Pig skin samples; (**B**) Human skin sample.

From the analysis of whole workflows, where the effects of different factors discussed above accumulate, the best total RNA quality and quantity values for domestic pig samples were achieved with workflows which comprised sample collection into APTR, BME + LB or as dry biopsy and homogenizing in BME + LB applying Fastprep D tubes (WF6, WF7 and WF8), and workflows which comprised sample collection into APTR and latter homogenizing in QIAzol or collecting and homogenizing in BME + LB applying GentleMACS M tubes (WF10 and WF14) (Fig. 1A, Table 2S in Supplementary Materials). Therefore, the workflows comprising sample collection and homogenizing in BME + LB or QIAzol and applying different homogenizers (WF7_D, WF14_M and WF3_D and WF11_M, respectively) were tested with human samples.

**Figure 2:**
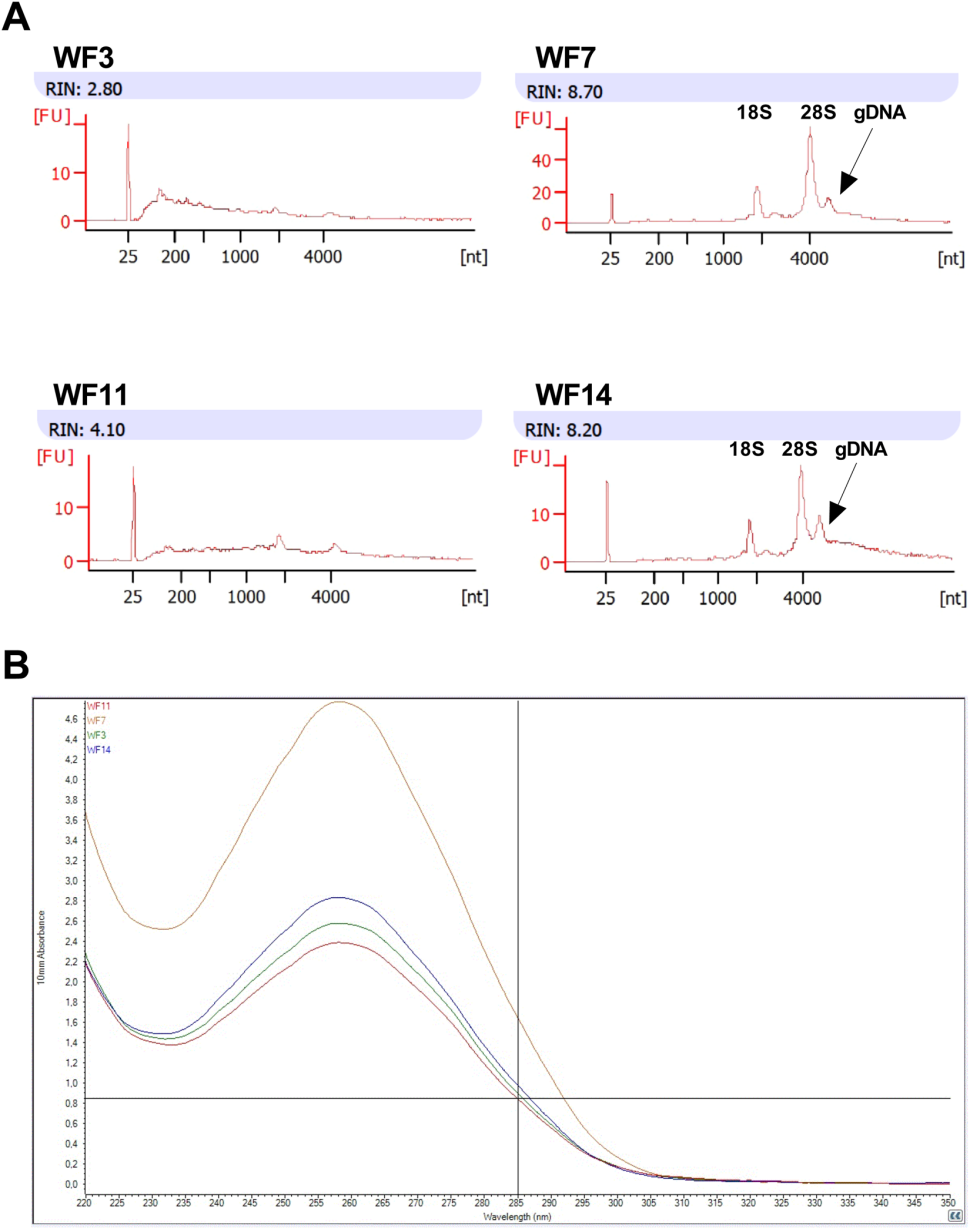
RNA quality values from human skin derived RNA samples. (**A**) RNA Integrity Numbers (RIN) obtained by applying the Agilent 2100 Bioanalyzer and RNA 6000 Nano kit. (**B**) The RNA absorbance spectrum measured with NanoDrop spectrophotometer.

With human skin tissue it was found that by applying BME + LB for sample collection, storage and homogenization, it was possible to gain RNA with higher quality and quantity, but with higher DNA contamination, compared to QIAzol (Figs. 2 and 6S in Supplementary Materials). Similar to pig skin, only GentleMACS with M tube was able to fully homogenize human skin samples. However, it was more efficient for human skin as all samples were fully homogenized after only one cycle. The Fastprep with D tubes was not able to fully homogenize and resulted in cloudy homogenizing buffer with intact skin pieces. Additionally, as with domestic pig skin, the quality of human skin RNA extracted applying Fastprep with D tubes or GentleMACS with M tubes did not differ significantly (Fig. 7S in Supplementary Materials).

Analysis of the four workflows applied to human skin (Fig. 1B), demonstrated that, in general, higher total RNA quality and quantity values are achieved with WF7_D and WF14_M, which include biopsy collection directly into RLT lysis buffer containing additional beta-mercaptoethanol (BME + LB), freezing within an hour at −20 °C and transferring to −80 °C within a week, and finally homogenizing with the Fastprep homogenizer with D tubes or with the GentleMACS Dissociator with M tubes, respectively, prior the standard RNA extraction protocol of Qiagen RNeasy Fibrous Tissue Mini kit. However, similar to domestic pig skin experiments, the DNA contamination with WF7_D was slightly higher than with WF14_M.

The expression patterns of four reference genes (*HPRT1, OSBP, PGK1, SDHA*) were similar to each other. Furthermore, the ΔCq values were lower for samples with higher RIN (Fig. 8S). For example, when analysing *HPRT1* gene, in case of BME + LB and QIAzol WFs, the average ΔCq values were −0.1 and 1.4, and the RIN values were 8.4 and 2.4, respectively. Thus, qPCR confirmed the higher quality of samples collected into BME + LB compared to samples collected into QIAzol (WF7 and WF14 versus WF3 and WF11, respectively).

### The suitability of domestic pig skin as a model of human skin

Domestic pig skin was considered as the best choice as a model for human skin based on information available in the literature. The findings of our study supported this choice, as the four WFs tested yielded similar results for human as for pig skin samples. The GentleMACS Dissociator was more efficient for tissue homogenizing compared to the Fastprep-24 Instrument, and the BME + LB solution resulted in RNA samples of higher quality and quantity but also higher DNA contamination. Additionally, in most cases (two pigs out of three and all human samples) there was no correlation between the input tissue weight and RNA yield (Fig. 10SA in Supplementary Materials). Furthermore, when using all the quantity (RNA yield) and quality (DNA contamination%, OD A260/280 and A260/230 and RIN) values as inputs to a principal component analysis (PCA), there were no differences seen between the skin sample groups of domestic pig and human (Fig. 10SB in Supplementary Materials). However, there was one major difference between human and pig skin samples: QIAzol was found to be ineffective as a sample stabilizing solution during sample collection with human skin tissue, despite being effective to keep RNA intact in case of pig skin.

## Discussion

In the present study we were successful in applying domestic pig skin as model for analysing human skin regarding RNA extraction. Furthermore, we were able to find an optimal protocol among the twenty workflows tested for extracting high quality and quantity RNA from human skin.

Between the three domestic pigs (front limb), only in one case was there a significant correlation between the skin biopsy weight and the RNA yield. There was no correlation between the skin biopsy weight and the RNA yield in the case of human samples. Thus, we cannot expect higher RNA yield from larger skin biopsies. In previous studies, the correlation between tissue weight and RNA yield has been controversial. In the case of human (upper thigh or arm) and mouse (back) skin tissues, a linear relationship has been demonstrated^20^, while another study found no such correlation when analysing human cleft lip skin tissue^21^. The lack of correlation between tissue input and RNA output and the difference between individuals in the case of pigs showed that other factors might affect the RNA yield in addition to tissue weight. The difference may be due to the unequal water content in tissues before extracting the RNA. Variation in skin water content can be caused by different age, sex (menstrual cycle in case of women), and environmental factors (such as water consumption prior to sample collection)^22–25^. In the present study, pigs were all immature females from the same age group and grew up together in same environmental conditions. However, in the case of human samples, the patients’ age, sex and body areas were distinct. Additionally, pathological skin conditions such as irritated and inflammatory skin^26^ tend to be associated with higher RNA expression levels. This is also true in the case of lesional psoriasis skin that yields a higher quantity of RNA compared to unlesional skin obtained from the same patients (unpublished observations by Ene Reimann).

Additionally, the Qiagen RNeasy Fibrous Tissue Mini kit handbook states that incomplete lysis of the starting material will decrease the RNA yield from the sample (RNeasy Fibrous Tissue Handbook, 10/2010). In our experiment, we had incomplete tissue homogenization in most cases and thus probably also insufficient lysis of the samples. However, in the case of six human samples fully homogenized by the GentleMACS Dissociator, there was still no correlation between tissue input and RNA yield (data not shown). Furthermore, it is often complicated to measure the weight of biopsies, and thus the amount of the sample applied to the spin column may overload it, which is another cause for decreased RNA yield and also quality (RNeasy Fibrous Tissue Handbook, 10/2010). If we analysed only the samples which had weight a quarter lower than the columns’ capacity, there was no correlation between the sample input and RNA yield or quality (OD rates and RIN) values (data not shown). These data support the suggestions from previous paragraph that the RNA output is not only affected by tissue input and homogenizing efficiency, but also by other factors which may have been specific to the sample.

Compared to collecting skin samples into APTR or QIAzol with delayed freezing, sample collection as dry biopsies with immediate freezing or into BME + LB with delayed freezing resulted in RNA with higher quality and quantity values. QIAzol is known to contain phenol and guanidine thiocyanate (QIAzol Handbook, 01/2009), and thus there may have been some contamination even after column purification. However, in case of APTR, precise information about the ingredients is not available, and the cause of lower purity values cannot be suggested. We found no previous literature to compare with our findings. Thus, according to our results, skin samples collected as dry biopsy or into BME + LB should be preferred to sample collection into APTR or QIAzol.

Our attempt to make the domestic pig skin tissue more fragile and susceptible to mechanical homogenization by applying combined hyaluronidase-collagenase digestion was not successful, as there was no significant difference in homogenizing efficiency between digested and not digested samples. Previously, collagenase treatment has been successfully applied to extract fibroblasts^27^ or tumour lymphocytes^28^ from human skin biopsies. Furthermore, it has been suggested that combining collagenase and hyaluronidase digestion prior to human skin homogenization would be advantageous (sisweb.com, 05/2009)), however, there is no information regarding the effect on homogenizing efficiency. In the case of porcine tissue, overnight incubation was conducted to induce dendritic cell migration^29^ from skin biopsy. However, at the time of this study, no literature was available regarding treating pig skin enzymatically prior to homogenization.

Additionally, the purpose of the enzymatic digestion was to evaluate whether it improves the homogenizing efficiency, and thus we did not add RNase inhibitors to our samples. Thus, obtaining highly degraded RNA from enzymatically treated samples was an expected result. However, if, for some reason, enzymatic digestion at 37 °C is needed, RNase inhibitors are strongly recommended^30,31^.

The bead motion-based Fastprep-24 Instrument was incapable of fully homogenizing skin tissues, although the manufacturer’s suggested homogenizing program was applied^7^. However, the stator and rotor based GentleMACS Dissociator was able to fully homogenize almost half of the pig skin biopsies and all the human skin biopsies. The RNA quality and quantity were nearly identical between these two homogenizing strategies. From previous literature, there are data available regarding comparison between different types of homogenizers, including these two, but not specifically on skin^32,33^. Thus, when relying on the information received from the present study, both the Fastprep-24 Instrument and the GentleMACS Dissociator might be considered suitable for homogenizing skin tissue.

Analysis of human skin experimental data confirmed the findings previously obtained from the domestic pig skin experiments. Applying WF7_D or WF14_M, it was possible to gain RNA with better quality and quantity, but with higher DNA contamination. Furthermore, for skin samples, the on-column DNase digestion seems to be insufficient for full digestion of the genomic DNA, and thus other strategies such as post-RNA-extraction DNase treatment should be considered. Although in the literature, there were no studies confirming our findings, we expected to obtain these results, as human and pig skin have similar characteristics in various aspects, but most importantly in morphology^11–19^. The results presented here showed that the domestic pig samples were indeed applicable as a model tissue for finding potentially good workflows for human skin experiments. Furthermore, applying pig skin tissue helped us to achieve the results with less human tissue.

In conclusion, we demonstrated that domestic pig can be used as a model for testing the recovery of RNA from human skin. Utilizing pig skin tissue helped to exclude less efficient strategies prior to the testing of the particular methods on human skin biopsies. We were able to find the optimal workflow for extracting RNA from human skin biopsies. Furthermore, skin tissue collection directly into lysis buffer proved to be an efficient strategy and that could be used instead of collecting samples as dry biopsies. The enzymatic digestion did not increase the homogenization efficiency and, in fact, degraded the RNA. The rotor and stator-based homogenizer was more efficient than the bead motion-based homogenizer for skin tissue homogenization, however, the RNA output after purification was rather similar. Nevertheless, thirteen out of twenty workflows in the case of domestic pig skin resulted in average RIN > 7, and thus could be considered as potential strategies for further RNA expression analysis.

## Materials and Methods

To analyse how different sample collection/storage, enzymatic treatment and homogenization methods work together, several commonly used strategies were analysed (Table 1). Below, there is general information about the applied materials and methods, however, the detailed protocols are reachable as supplementary material (Fig. 1S in Supplementary Materials).

### Sample collection and storage

Collecting domestic pig skin applying method described below does not require an ethical approval. In case of human subjects, the Ethical Review Committee on Human Research of the University of Tartu approved the study protocols and informed consent forms. All participants signed written informed consent.

Domestic pig (mixed from multiple breeds such as Yorkshire, Landrace and Duroc) skin from the front limbs of three female piglets was collected immediately after slaughter. The pig samples were collected free of charge in Tartu County (Estonia) slaughterhouse owned by OÜ Rotaks-R. The skin was cut so that it contained the layers of epidermis, dermis and minimum amount of subcutis. Four different sample collection/storage strategies were tested – samples were (1) collected as dry biopsies and immediately frozen on dry ice; (2) collected into Allprotect Tissue Reagent (APTR; Qiagen, Hilden, Germany), kept overnight at +4 °C and then transferred to −80 °C; (3) collected into QIAzol Lysis reagent (QIAzol) or (4) into RTL lysis buffer from RNeasy Fibrous Tissue Mini kit, containing beta-mercaptoethanol (BME + LB) (both from Qiagen, Hilden, Germany) and frozen at −80 °C 2–3 hours after collection.

All six human subjects in the study were Caucasians living in Estonia and were recruited from among the patients at the dermatologic outpatient clinic. The skin tissues collected for the study were derived from the edges of the unpigmented skin areas from a birthmark removal surgery (two pieces per patient). Among six patients there were two men and four women, the average age was 43 years (±SD 13) and the body areas were abdomen, thigh, chest, arm, and side of a trunk. For human, only two sample collection and storage strategies were used – samples were collected into QIAzol or BME + LB, frozen at −20 °C ~1 hour after collection and transferred to −80 °C within a week after collection (best possible conditions available in clinic).

All domestic pig and human samples were stored at −80 °C for two months before RNA extraction. All the skin pieces were weighed prior homogenizing and for the following procedures full biopsies were used. Each workflow had three biological replicates.

### Enzymatic digestion

The enzymatic digestion was tested only on domestic pig samples. For that, the whole biopsies were treated at 37 °C for 2 hours with combined collagenase and hyaluronidase solution (both from Sigma-Aldrich, Darmstadt, Germany). After that, samples were centrifuged, the supernatant was removed, and samples were transferred into homogenizing tubes containing the homogenizing buffer (QIAzol or BME + LB) and homogenized immediately.

### Homogenizing

Two homogenizing solutions (QIAzol or BME + LB), two instruments with three different types of disposable tubes (bead motion-based Fastprep-24 instrument with lysing matrix D (ceramic beads) or S tubes (metal beads) (MP Biomedicals, California, USA) and stator and rotor-based GentleMACS Dissociator with M tubes (Miltenyi Biotec, Bergisch Gladbach, Germany) were tested for homogenizing domestic pig samples. In case of human samples, two homogenizing solutions (QIAzol or BME + LB) and Fastprep-24 instrument with lysing matrix D tubes or GentleMACS Dissociator with M tubes was applied. The homogenizing efficiency was evaluated visually and termed as “fully homogenized” if no intact skin piece was seen. After homogenizing, RNA purification was carried out immediately according to RNA purification protocol.

### RNA purification

For samples homogenized in QIAzol, chloroform was added, and tubes were shaken vigorously and centrifuged, separating the preparation into three phases. The upper aqueous phase, containing the RNA, was transferred into a new tube and ethanol was added. Then the column purification combined with DNase treatment was conducted according to manufacturer’s protocol, applying RNeasy Fibrous Tissue Mini kit with RNase-Free DNase Set (Qiagen, Hilden, Germany).

For samples homogenized in BME + LB with additional nuclease free water, a standard protocol of RNeasy Fibrous Tissue Mini kit with RNase-Free DNase Set was applied, however, the proteinase K and absolute ethanol amount was adjusted to the BME + LB volume applied for homogenizing.

### RNA quantity and quality assessment

For measuring the quantity of extracted RNA and evaluating DNA contamination the Qubit 2.0. fluorometer with Qubit RNA HS Assay Kit or DNA HS Assay kit (Thermo Fischer Scientific, Waltham, USA) was applied, respectively. This method is able to clearly distinguish between DNA and RNA molecules and thus provides reliable information about RNA concentration and possible DNA contamination (User Guide: Qubit dsDNA HS Assay Kits, MAN0002326 | MP32851, Revision B.0), (User Guide: Qubit RNA HS Assay Kits, MAN0002327 | MP32852, Revision: A.0). For evaluating RNA integrity the Agilent 2100 Bioanalyzer and RNA 6000 Nano kit (Agilent Technologies, California, USA) was applied. Based on the entire electrophoretic trace of RNA sample, the RNA Integrity Number (RIN) is calculated by 2100 Expert Software and given as a numerical value (Agilent 2100 Bioanalyzer 2100 Expert User’s Guide, May 2005), (Agilent RNA 6000 Nano Kit Guide, 07/2013 G2938-90034 Rev. B) RIN = 7 is often taken as an RNA sample quality threshold for the suitability for expression analysis (Ion T RNA-Seq Kit v2 - User Guide. MAN0010654 (2017). A NanoDrop spectrophotometer (Thermo Fischer Scientific, Waltham, USA) was used to evaluate the purity of RNA samples. According to the Nanodrop manual the A260/280 ratio of ~2.0 and A260/230 ratio of 1.8–2.2 is generally accepted as “pure”; a low A260/280 ratio indicates the presence of protein, phenol or other contaminants that absorb strongly at or near 260 nm; similarly, a low A260/230 ratio indicates the presence of residual phenol, guanidine, magnetic beads, carbohydrates or proteins (NanoDrop Nucleic Acid Handbook, 11/2010).

### qPCR analysis

To additionally confirm the RNA quality at RNA expression level, we applied the frequently used qPCR method^34–36^. Although, it is possible to screen the whole transcriptome with arrays or sequencing platforms^37–39^, these approaches are still rather expensive. With qPCR it is possible to analyse both miRNA and mRNA expression, however, as we applied the protocols for which only RNA molecules longer than 200 bp are purified (RNeasy Fibrous Tissue Handbook, 10/2010), we analysed only mRNA expression with qPCR. In RNA samples with poor quality, the higher grade of molecules has degraded, which stands out in the results as higher Cq values, compared to samples with high quality. This effect can be seen due to the lower amount of cDNA molecules synthesized, as the process starts from the polyA tail, which is often damaged in degraded RNA molecules (Qiagen. qPCR: RNA Quality and Why It Matters, 2017).

Four reference genes (*HPRT1, OSBP, PGK1, SDHA*) previously used in different skin studies^40–43^ were applied to confirm the RNA quality derived from human samples – RNA samples with higher RIN values should have lower ΔCq values. In the case of all four genes, we have previously validated the even expression level between psoriasis patients lesional and unlesional skin and control skin applying RNA-seq and/or qPCR (data not published). We analysed both high-quality (RIN > 7) and lower-quality RNA samples (RIN < 7) to confirm that the expression pattern of selected genes is affected by the general RNA integrity values.

The qPCR experiments were conducted according to the MIQE guidelines^44^. 500 ng of total RNA was used with FIREScript RT cDNA Synthesis Mix (Solis BioDyne, Tartu, Estonia) for cDNA synthesis, according to the manufacturer’s protocol. cDNA was used as a template for qPCR using the Quantstudio 12k Flex Real-Time PCR system platform (Thermo Fisher Scientific Inc., CA, USA). The qPCR conditions for all four reference genes (for all genes the amplification efficiencies were 100% ± 10%) were the same. The qPCR was conducted in four replicates and with reaction volume 10 ul on 384 plate formats using EvaGreen qPCR Supermix (Solis BioDyne, Tartu, Estonia), final primer concentration 400 nM and cDNA input 2.5 ng (10 × dilution) per reaction. The qPCR program was as follows: hold stage −95 °C, 15 min; 40 cycles of PCR stage −95 °C, 20 sec; 60 °C, 20 sec; 72 °C, 20 sec, melt curve stage 95 °C, 20 sec; 60 °C, 20 sec; 95 °C, 20 sec. The pool of all twelve human RNA samples in equal amounts (ng) (CS_Hs) was used as a calibration sample on each PCR plate.

Cq values were taken as average from the four (or less) technical replicates. The formulas for ΔCq calculation was as follows: ΔCq = Cq_sample_ − Cq_Cs_Hs_. For correlation and comparative analysis, ΔCq was used.

### Data analysis

For data analysis and generation of graphs and figures, GraphPad Prism 6 (GraphPad Software, California, USA), Microsoft Excel and PowerPoint (Microsoft Corporation, Washington, USA) were applied. For evaluating the efficiency of the different strategies or whole workflows, all the quality and quantity values (RIN, total RNA amount, OD 260/280 and OD 260/230, and DNA contamination) were considered; however, the high total RNA amount and RIN values were considered critical for higher efficiency. In Figs. 1 and 3S–7S sample groups were ordered according to the RIN values, starting from the group with highest RIN value.

## Supplementary information

Supplementary Materials

## References

[1] Trost A (2007). Rapid, high-quality and epidermal-specific isolation of RNA from human skin. Exp. Dermatol..

[2] Mendez V (2011). A rapid protocol for purification of total RNA for tissues collected from pigs at a slaughterhouse. Genet. Mol. Res..

[3] Auer H (2014). The effects of frozen tissue storage conditions on the integrity of RNA and protein. Biotech. Histochem..

[4] Mutter GL (2004). Comparison of frozen and RNALater solid tissue storage methods for use in RNA expression microarrays. BMC Genomics.

[5] Lou JJ (2014). A review of room temperature storage of biospecimen tissue and nucleic acids for anatomic pathology laboratories and biorepositories. Clin. Biochem..

[6] Gardner H (2006). Gene profiling of scleroderma skin reveals robust signatures of disease that are imperfectly reflected in the transcript profiles of explanted fibroblasts. Arthritis Rheum..

[7] Berglund SR (2007). Optimized methodology for sequential extraction of RNA and protein from small human skin biopsies. J. Invest. Dermatol..

[8] Keermann M (2015). Transcriptional landscape of psoriasis identifies the involvement of IL36 and IL36RN. BMC Genomics.

[9] Reimann E (2012). The mRNA expression profile of cytokines connected to the regulation of melanocyte functioning in vitiligo skin biopsy samples and peripheral blood mononuclear cells. Hum. Immunol..

[10] Samadani AA (2015). RNA Extraction from Animal and Human's Cancerous Tissues: Does Tissue Matter?. Int. J. Mol. Cell Med..

[11] Jung EC, Maibach HI (2015). Animal models for percutaneous absorption. J. Appl. Toxicol..

[12] Simon GA, Maibach HI (2000). The pig as an experimental animal model of percutaneous permeation in man: qualitative and quantitative observations–an overview. Skin Pharmacol. Appl. Skin Physiol..

[13] Boudry I, Trescos Y, Vallet V, Cruz C, Lallement G (2008). Methods and models for percutaneous absorption studies of organophosphates. Pathol. Biol. (Paris).

[14] Debeer S (2013). Comparative histology and immunohistochemistry of porcine versus human skin. Eur. J. Dermatol..

[15] Gutierrez K, Dicks N, Glanzner WG, Agellon LB, Bordignon V (2015). Efficacy of the porcine species in biomedical research. Front. Genet..

[16] Avon SL, Wood RE (2005). Porcine skin as an *in-vivo* model for ageing of human bite marks. J. Forensic Odontostomatol..

[17] Branski LK (2008). A porcine model of full-thickness burn, excision and skin autografting. Burns.

[18] Oxenhandler RW, Adelstein EH, Haigh JP, Hook RR, Clark WH (1979). Malignant melanoma in the Sinclair miniature swine: an autopsy study of 60 cases. Am. J. Pathol..

[19] Mitra A (2015). Use of minipig skin biopsy model as an innovative tool to design topical formulation to achieve desired pharmacokinetics in humans. J. Pharm. Sci..

[20] Bruning O (2011). RNA isolation for transcriptomics of human and mouse small skin biopsies. BMC Res. Notes.

[21] Syazana MSN, Wan Sulaiman WA, Halim AS, Sarina S (2014). Skin Tissue Surface Morphology and Quality of RNA and Protein Extracted from Fresh and Stabilized Human Cleft Lip and Palate Tissue. Maced. J. Med. Sci..

[22] Reinoso RF, Telfer BA, Rowland M (1997). Tissue water content in rats measured by desiccation. J. Pharmacol. Toxicol. Methods.

[23] Raja MK, Raymer GH, Moran GR, Marsh G, Thompson RT (2006). Changes in tissue water content measured with multiple-frequency bioimpedance and metabolism measured with 31P-MRS during progressive forearm exercise. J. Appl. Physiol. (1985).

[24] Hashimoto-Kumasaka K, Takahashi K, Tagami H (1993). Electrical measurement of the water content of the stratum corneum *in vivo* and *in vitro* under various conditions: comparison between skin surface hygrometer and corneometer in evaluation of the skin surface hydration state. Acta Derm. Venereol..

[25] de Farias Pires T (2016). A population-based study of the stratum corneum moisture. Clin. Cosmet. Investig. Dermatol..

[26] Wong R (2004). Use of RT-PCR and DNA Microarrays to Characterize RNA Recovered by Non-Invasive Tape Harvesting of Normal and Inflamed Skin. J. Invest. Dermatol..

[27] Wang H, Van Blitterswijk CA, Bertrand-De Haas M, Schuurman AH, Lamme EN (2004). Improved enzymatic isolation of fibroblasts for the creation of autologous skin substitutes. In Vitro Cell Dev. Biol. Anim..

[28] Novelli M (2000). Collagenase digestion and mechanical disaggregation as a method to extract and immunophenotype tumour lymphocytes in cutaneous T-cell lymphomas. Clin. Exp. Dermatol..

[29] Marquet F (2014). Pig skin includes dendritic cell subsets transcriptomically related to human CD1a and CD14 dendritic cells presenting different migrating behaviors and T cell activation capacities. J. Immunol..

[30] Ramalho AS (2004). Methods for RNA extraction, cDNA preparation and analysis of CFTR transcripts. J. Cyst. Fibros..

[31] Earl CC, Smith MT, Lease RA, Bundy BC (2018). Polyvinylsulfonic acid: A Low-cost RNase inhibitor for enhanced RNA preservation and cell-free protein translation. Bioengineered.

[32] Poggel, C., Adams, T., Langhammer, A. & Bosio, A. *Robust and reproducible automated tissue homogenization*.

[33] Goldberg S (2015). Mechanical/physical methods of cell distribution and tissue homogenization. Methods Mol. Biol..

[34] Jaguszewski M (2014). A signature of circulating microRNAs differentiates takotsubo cardiomyopathy from acute myocardial infarction. Eur. Heart J..

[35] Padhi BK, Singh M, Rosales M, Pelletier G, Cakmak S (2018). A PCR-based quantitative assay for the evaluation of mRNA integrity in rat samples. Biomol. Detect. Quantif..

[36] Hantzsch M (2014). Comparison of whole blood RNA preservation tubes and novel generation RNA extraction kits for analysis of mRNA and MiRNA profiles. PLoS One.

[37] Vartanian K (2009). Gene expression profiling of whole blood: comparison of target preparation methods for accurate and reproducible microarray analysis. BMC Genomics.

[38] Malone JH, Oliver B (2011). Microarrays, deep sequencing and the true measure of the transcriptome. BMC Biol..

[39] Wang Z, Gerstein M, Snyder M (2009). RNA-Seq: a revolutionary tool for transcriptomics. Nat. Rev. Genet..

[40] Bar M, Bar D, Lehmann B (2009). Selection and validation of candidate housekeeping genes for studies of human keratinocytes–review and recommendations. J. Invest. Dermatol..

[41] He JQ (2008). Selection of housekeeping genes for real-time PCR in atopic human bronchial epithelial cells. Eur. Respir. J..

[42] Turabelidze A, Guo S, DiPietro LA (2010). Importance of housekeeping gene selection for accurate reverse transcription-quantitative polymerase chain reaction in a wound healing model. Wound Repair Regen..

[43] Durrenberger PF (2012). Selection of novel reference genes for use in the human central nervous system: a BrainNet Europe Study. Acta Neuropathol..

[44] Bustin SA (2009). The MIQE guidelines: minimum information for publication of quantitative real-time PCR experiments. Clin. Chem..

