# Supplementary material for: Identification of an optimal method for extracting RNA from human skin biopsy, using domestic pig as a model system
Source: Sci Rep. 2019 Dec 27;9:20111. doi: 10.1038/s41598-019-56579-5 (PMC6934780; doi:10.1038/s41598-019-56579-5)
Supplement: Supplementary file 1 — Supplementary Materials [file 41598_2019_56579_MOESM1_ESM.pdf]

SUPPLEMENTARY MATERIALS

TITLE PAGE

Identification of an optimal method for extracting RNA from human skin biopsy, using domestic pig as a model system.

Ene Reimann[1]*, Kristi Abram[2,3], Sulev Kõks[4,5], Külli Kingo[2,3] and Alireza Fazeli[1,6]

[1]Department of Pathophysiology, University of Tartu, Tartu, Estonia;
[2]Department of Dermatology, University of Tartu, Tartu, Estonia;
[3]Clinic of Dermatology, Tartu University Hospital, Tartu, Estonia;
[4]Centre for Comparative Genomics, Murdoch University, Perth 6150, Australia;
[5]The Perron Institute for Neurological and Translational Science, Perth 6009, Australia;
[6]Academic Unit of Reproductive and Developmental Medicine, Department of Oncology and Metabolism, The Medical School, University of Sheffield, Sheffield, United Kingdom
* Corresponding author

e-mail addresses of authors:
Ene Reimann: ene.reimann@ut.ee
Kristi Abram: kristi.abram@kliinikum.ee
Sulev Kõks: sulev.koks@ut.ee
Külli Kingo: kylli.kingo@kliinikum.ee
Alireza Fazeli: alireza.fazeli@ut.ee

MATERIALS AND METHODS

Sample collection and storage

Domestic pig samples

Skin from front limb of three female piglets (mixed from multiple breeds such as Yorkshire, Landrace and Duroc) was collected immediately after slaughter. The pig samples were collected free of charge in Tartu County (Estonia) slaugtherhouse owned by OÜ Rotaks-R. Collecting skin samples like this does not require an ethical approval. The skin was removed from the limb, washed with 70 % ethanol and cut into approximately 3 x 3 mm pieces applying single-use scalpels and tweezers. The skin was cut so that it contained the layers of epidermis, dermis and minimum amount of subcutis. Altogether 40 skin pieces per animal was collected.

Four different sample collection/storing strategies were tested with pig skin samples. (1) the samples were collected as dry biopsies – after removing the tissue it was transferred into empty 1.5 ml tube (Eppendorf LoBind microcentrifuge tube, Sigma-Aldrich, Darmstadt, Germany) and immediately frozen on dry ice. (2) The samples were collected into tubes containing ~ 500 µl of Allprotect Tissue Reagent (APTR; Qiagen, Hilden, Germany). The samples in APTR were kept overnight at + 4 °C and then transferred to – 80 °C. (3) The samples were collected into tubes containing 1 ml of QIAzol Lysis reagent (QIAzol) or (4) samples were collected into 433.3 ml of RTL lysis buffer from RNeasy Fibrous Tissue Mini kit, containing beta-mercaptoethanol (BME + LB) (both solutions from Qiagen, Hilden, Germany). The samples in QIAzol and BME + LB were frozen at – 80 °C 2-3 hours after collection.

All samples were stored at – 80 °C for two months before the RNA extraction. All the skin pieces were weighed prior enzymatic digestion or homogenizing and for the following procedures, the full biopsies were used. Each workflow had three biological replicates.

Human samples

In case of human samples, the Ethical Review Committee on Human Research of the University of Tartu approved the study protocols and informed consent forms. All participants signed written informed consent. All six human subjects in the study were Caucasians living in Estonia and were recruited from among the patients at the

dermatologic outpatient clinic. The skin tissues collected for the study were derived from the edges of the unpigmented skin areas from a birthmark removal surgery (two pieces per patient). No additional information such as patient's age or sex was received from the clinic.

For human, only two sample collection and storage strategies were used - human samples were collected into 1.5 ml tubes (Eppendorf LoBind microcentrifuge tube, Sigma-Aldrich, Darmstadt, Germany) containing 1 ml QIAzol or 433.3 ml of BME + LB, frozen at – 20 °C ~ 1 hour after collection and transferred to – 80 °C within a week after collection (best possible conditions available in clinic).

All samples were stored at – 80 °C for two months before the RNA extraction. All the skin pieces were weighed prior homogenizing and for the following procedures full biopsies were used. Each workflow had three biological replicates.

Enzymatic digestion

The enzymatic digestion was tested only on domestic pig samples. For that, first the fresh collagenase/hyaluronidase working solution was prepared – collagenase (final concentration 125 U/ml (Sigma-Aldrich cat no: C0130, Darmstadt, Germany)) and hyaluronidase (final concentration 100 U/ml (Sigma-Aldrich, cat no: H3506, Darmstadt, Germany)) was diluted into Hank's Balanced Salt Solution (HBBS, Naxo OÜ, cat no: 2008, Tartu, Estonia). Enzymatic digestion was conducted only for dry biopsy and APTR samples, and in case of APTR samples the tissue was washed once in 1 ml HBSS buffer prior to treatment. For enzymatic digestion 1 ml of enzyme working solution was added to the whole biopsy in a 1.5 ml tube (Eppendorf LoBind microcentrifuge tube, (Sigma-Aldrich, Darmstadt, Germany) and incubated at 37 °C and at 500 rpm for 2 hours applying the thermostat shaker. After that samples were centrifuged at room temperature (RT; 15–25 °C) at 1000 x g for 5 min; thereafter the supernatant was removed, and samples were transferred into homogenizing tubes containing the homogenizing buffer (QIAzol or BME + LB) and homogenized immediately.

Homogenizing

Two homogenizing solutions (QIAzol or BME + LB), two instruments with three different tubes (Fastprep-24 instrument with lysing matrix D or S tubes (cat no:

116913050 and 116925050, respectively; MP Biomedicals, California, USA) and GentleMACS Dissociator with M tubes (cat no: 130-093-236; Miltenyi Biotec, Bergisch Gladbach, Germany)) were tested for homogenizing domestic pig samples. In case of human samples two homogenizing solutions (QIAzol or BME + LB) and Fastprep-24 instrument with lysing matrix D tubes or GentleMACS Dissociator with M tubes was applied. Skin samples were homogenized in 1 ml QIAzol reagent or 433.3 µl BME + LB buffer with 852 µl of nuclease free water (nuclease free water was added prior homogenizing as the Fastprep 2 ml tubes require at least 800 µl of homogenizing buffer). Samples collected into APTR or dry biopsy samples (with or without prior enzymatic digestion) were transferred into homogenizing tube containing new homogenizing buffer. Samples collected directly into QIAzol or BME + LB, were transferred into homogenizing tube together with the buffer they were initially stored. In case of Fastprep homogenizing the program was as follows: speed 6.0, time 40 seconds, 5 cycles. The dry ice was added into the rotor between the cycles to cool the system and samples. In case of GentleMACS Dissociator the program RNA_02.01 was applied; altogether two cycles were used, and samples were placed on wet ice between the cycles. The homogenizing efficiency was evaluated visually and termed as "fully homogenized" if no intact skin piece was seen. After homogenizing, the samples were transferred into new 2 ml tubes (Eppendorf LoBind microcentrifuge tube, Sigma-Aldrich, Darmstadt, Germany) and immediately proceed according to RNA purification protocol.

RNA purification

Samples homogenized in QIAzol

After homogenization the samples were placed at RT for 5 min, after which 0.2 ml of chloroform per 1 ml QIAzol was added and samples were shaken vigorously for 15 sec. The tubes containing the homogenates were placed at RT for 2–3 min and after that centrifuged at 12,000 x g for 15 min at 4 °C. After centrifugation, samples were separated into three phases: an upper, colourless, aqueous phase is containing RNA and is approximately 60% of the volume of the QIAzol. The upper aqueous phase was transferred into a new 1.5 ml tube and 1 x volumes of freshly prepared 70 % ethanol was added and mixed by pipetting. After that the column purification combined with DNase treatment was conducted according to manufacturer's protocol applying RNeasy Fibrous

Tissue Mini kit with RNase-Free DNase Set (cat no: 74704 and 79254, respectively; Qiagen, Hilden, Germany). The final elution volume for all samples was 50 µl.

Samples homogenized in BME + LB buffer

In case of samples homogenized in BME + LB buffer with additional nuclease free water, 14.4 µl of proteinase K solution from RNeasy Fibrous Tissue Mini kit was added and samples were incubated at 55 °C for 10 min. After that samples were centrifuged at RT for 3 min at 10,000 x g after which a small pellet of tissue debris was formed, sometimes accompanied by a thin layer or film on top of the supernatant. The supernatant (approximately 1300 µl) was transferred into a new 2.0 ml tube, avoiding transferring any of the pellet and 0.5 x volumes of absolute ethanol (100%) was added to the cleared lysate and mixed by pipetting. After that the column purification combined with DNase treatment was conducted according to manufacturer's protocol applying RNeasy Fibrous Tissue Mini kit with RNase-Free DNase Set. The final elution volume for all samples was 50 µl.

RNA quantity and quality assessment

For measuring the quantity of extracted RNA, the Qubit 2.0. fluorometer and Qubit RNA HS Assay Kit (Thermo Fischer Scientific, Waltham, USA) was applied. Depending on the sample concentration 1 µl of stock RNA or 1 µl of 10 times dilution was used as input. For measuring the DNA contamination Qubit 2.0 fluorometer with DNA HS Assay kit (Thermo Fischer Scientific, Waltham, USA) was applied using 1 µl of stock RNA. The advantage of this method is, that it is able to clearly distinguish between DNA and RNA molecules and thus provide reliable information about RNA concentration and possible DNA contamination. [1,2]

For evaluating the RNA integrity Agilent 2100 Bioanalyzer and RNA 6000 Nano kit (Agilent Technologies, California, USA) was applied. Depending on the sample concentration 1 µl of stock RNA or 1 µl of 10 times dilution was used as input. Based on the entire electrophoretic trace of RNA sample, the RNA Integrity Number (RIN) is calculated by 2100 Expert Software and given as a numerical value. [3,4] RIN = 7 is often taken as a RNA sample quality threshold for the suitability for expression analysis.

NanoDrop spectrophotometer (Thermo Fischer Scientific, Waltham, USA) was used with 2 μl of stock sample to evaluate the purity RNA sample. According to Nanodrop instrument manual the A260/280 ratio of ~ 2.0 and A260/230 ratio of 1.8-2.2 is generally accepted as "pure", as low A260/280 ratio indicates the presence of protein, phenol or other contaminants that absorb strongly at or near 260 nm and low A260/230 ratio indicates the presence of residual phenol, guanidine, magnetic beads, carbohydrates or proteins. [5]

qPCR analysis

To additionally confirm the RNA quality at RNA expression level, we applied the frequently used qPCR method. [6-8] Although, it is possible to screen the whole transcriptome with arrays or sequencing platforms [9-11], these approaches are still rather expensive. With qPCR it is possible to analyse both miRNA and mRNA expression; however, as we applied the protocols, where only RNA molecules longer than 200 bp are purified [12], we analysed only mRNA expression with qPCR. In RNA samples with poor quality the higher grade of molecules has degraded, which stands out in the results as higher Cq values, compared to samples with high quality. This effect can be seen due to the lower amount of cDNA molecules synthesized, as the process starts from polyA tail, which is often damaged in degraded RNA molecules. [13]

Four reference genes (*HPRT1, OSBP, PGK1, SDHA*) previously used in different skin studies [14-17] were applied to confirm the RNA quality derived from human samples – RNA samples with higher RIN values should have lower ΔCq values. In case all four genes we have previously validated the even expression level between psoriasis patients lesional and unlesional skin and control's skin applying RNA-seq and/or qPCR (data not published). We analysed both high-quality (RIN > 7) and lower-quality RNA samples (RIN < 7) to confirm, that the expression pattern of selected genes is affected by the general RNA integrity values.

Primers were designed using Primer3 software v4.1.0 [18]. Detailed information for the primers used in the qPCR experiments is presented in Table 3S. *In silico* primer specificity was screened with GenomeTester 1.3 [19]. The specificity was also confirmed by melting curve analysis and Sanger sequencing.

The qPCR experiments were conducted according to the MIQE guidelines [20]. 500 ng of total RNA was used with FIREScript RT cDNA Synthesis Mix (Solis BioDyne, Tartu, Estonia) for cDNA synthesis, according to the manufacturer's protocol. The PCR inhibition was tested with different cDNA dilutions and ten times dilution (final amount in PCR reaction is 2.5 ng) was applied for further experiments. cDNA was used as a template for qPCR in Quantstudio 12k Flex Real-Time PCR system platform (Thermo Fisher Scientific Inc., CA, USA). For primer validation the pool of all twelve human RNA samples (CS_Hs) in equal amounts (ng) was applied. The CS_Hs was also used as calibration sample on each PCR plate.

The qPCR conditions for all four reference genes (for all genes the amplification efficiencies were 100 % ± 10 %) were the same. The qPCR was conducted in four replicates and with reaction volume 10 ul on 384 plate formats using EvaGreen qPCR Supermix (Solis BioDyne, Tartu, Estonia), final primer concentration 400 nM and cDNA input 2.5 ng (10 x dilution) per reaction. The qPCR program was as follows: hold stage – 95 °C, 15 min; 40 cycles of PCR stage – 95 °C, 20 sec; 60 °C, 20 sec; 72 °C, 20 sec, melt curve stage 95 °C, 20 sec; 60 °C, 20 sec; 95 °C, 20 sec. The information about the qPCR validation can be found in Table 4S and Figure 9S.

Cq values were taken as average from the four (or less) technical replicates. The formulas for ΔCq calculation was as follows: $\Delta Cq = Cq_{sample} - Cq_{Cs\_Hs}$. For correlation and comparative analysis ΔCq was used.

Data analysis

In case of domestic pig and human samples, the average values of input (tissue weight) and output data (RNA quality and quantity values) from three biological replicates were calculated and used for further analysis and generating figures and graphs.

The first step in further data analysis was to evaluate whether the weight of skin tissue and amount of extracted RNA were correlated. For that Spearman's correlation was applied as neither the domestic pig nor human samples passed the normality test (D'Agostino and Pearson omnibus normality test). For further normalizing the total RNA amount was adjusted according to the weight of biopsy; the following formula was applied: Total RNA amount/Biopsy weight. For evaluating DNA contamination in

extracted RNA samples as percentages, the following formula was applied: total DNA amount/(total RNA amount + total DNA amount)*100 %.

For data analysis and generating graphs and figures GraphPad Prism 6 (GraphPad Software, California, USA) and Microsoft Excel and PowerPoint (Microsoft Corporation, Washington, USA) were applied. For statistical analysis the Mann-Whitney test was applied, if the sample groups did not follow the normal distribution or sample size was too small. If sample groups did follow the normal distribution Unpaired t test was applied.

In present study for evaluating the efficiency of the different strategies or whole workflows, all the quality and quantity values (RIN, total RNA amount, OD 260/280 and OD 260/230, and DNA contamination) were considered; however, the high total RNA amount and RIN values were considered critical for higher efficiency. In Figures 1 and 3S-7S sample groups were ordered accoding to the RIN values, starting from the group with highest RIN value.

For the principal component analysis, the RNA quantity (total amount of RNA (μg)) and quality values (OD 280/260, OD 230/260, RIN, DNA contamination %) of domestic pig and human samples were applied. Data was analysed and visualized as standardized (z-score (sample value – average sample value)/standard deviation)) values. ggplot2 package [21] were used for data visualization. Principal components were calculated using *prcomp()* function from the Stats package [22].

Table 1S. The results of factors observed in the study. Groups containing term "_Enz" includes also the data from enzymatically digested samples.

Factor type	Factor	Biopsy weight (mg)	Total RNA amount (μg)	DNA contamination (%)	NanoDrop 260/280	NanoDrop 260/230	Bioanalyzer RIN
**Pig samples**							
**Sample collection**	Dry Biopsy_Enz	54.8 (16.5)	13.7 (13.5)	10.1 (3.7)	2.1 (0.01)	1.7 (0.3)	7.0 (2.8)
	Dry Biopsy	51.3 (21.5)	22.6 (14.4)	10.8 (4.1)	2.1 (0.01)	1.8 (0.4)	9.0 (0.4)
	APTR_Enz	31.1 (14.1)	6.1 (5.5)	14.1 (11.4)	2.1 (0.10)	1.4 (0.6)	5.4 (3.6)
	APTR	29.5 (13.5)	6.7 (3.7)	12.7 (4.1)	2.0 (0.03)	1.6 (0.5)	8.9 (0.6)
	QIAzol	49.8 (11.5)	14.3 (15.0)	10.3 (3.9)	2.0 (0.02)	1.7 (0.6)	8.5 (0.9)
	BME + LB	56.6 (12.8)	18.3 (10.0)	11.7 (3.4)	2.1 (0.01)	1.9 (0.2)	8.9 (0.3)
**Homogenizing buffer**	QIAzol_Enz	37.3 (16.3)	9.2 (10.4)	9.2 (4.2)	2.0 (0.04)	1.5 (0.4)	6.5 (3.2)
	QIAzol	39.2 (16.9)	9.7 (11.4)	10.7 (4.0)	2.0 (0.01)	1.6 (0.4)	8.7 (0.7)
	BME + LB_Enz	42.4 (18.5)	10.5 (10.0)	15.7 (11.1)	2.1 (0.09)	1.6 (0.6)	6.6 (3.4)
	BME + LB	45.3 (18.2)	15.1 (9.8)	12.6 (3.6)	2.1 (0.01)	1.8 (0.4)	8.9 (0.5)
**Homogenizing instrument and tube**	Fastprep D_Enz	44.3 (18.4)	8.7 (8.6)	14.9 (12.5)	2.0 (0.04)	1.6 (0.4)	6.6 (3.3)
	Fastprep D	43.7 (18.3)	11.9 (9.3)	12.2 (4.3)	2.1 (0.02)	1.8 (0.3)	8.9 (0.4)
	Fastprep S_Enz	36.0 (18.2)	7.0 (7.1)	12.8 (7.7)	2.1 (0.13)	1.3 (0.6)	6.3 (3.6)
	Fastprep S	41.5 (19.7)	9.4 (7.6)	11.7 (4.7)	2.0 (0.02)	1.5 (0.4)	8.7 (0.7)
	GentleMACS M_Enz	37.9 (15.3)	14.5 (13.1)	10.8 (2.6)	2.0 (0.03)	1.8 (0.4)	7.5 (2.7)
	GentleMACS M	38.9 (15.4)	16.7 (14.2)	11.2 (2.5)	2.1 (0.02)	1.8 (0.4)	8.8 (0.8)
**Human samples**							
**Sample collection/ homogenizing buffer**	QIAzol	52.5 (23.3)	4.4 (1.1)	8.3 (2.0)	2.1 (0.01)	1.6 (0.22)	2.9 (0.7)
	BME + LB	78.8 (34.0)	7.3 (3.3)	13.6 (2.0)	2.1 (0.01)	1.8 (0.26)	8.4 (0.3)
**Homogenizing instrument and tube**	Fastprep D	81.7 (39.4)	6.9 (4.0)	10.6 (4.4)	2.1 (0.01)	1.7 (0.28)	5.6 (3.4)
	GentleMACS M	47.7 (12.4)	4.8 (1.0)	11.3 (2.6)	2.1 (0.01)	1.6 (0.29)	5.7 (2.8)

Table 2S. The RNA quantity and quality derived from different workflows. All values are presented as average (standard deviation) calculated from three biological replicates.

Workflow name	Biopsy weight (mg)	Total RNA amount (μg)	DNA contamination (%)	NanoDrop 260/280	NanoDrop 260/230	Bioanalyzer RIN
**Pig samples**						
WF1_D	39.3 (19.9)	4.2 (1.6)	5.2 (1.5)	2.0 (0.05)	1.1 (0.53)	2.3 (0.1)
WF1_S	24.7 (7.5)	3.4 (1.6)	4.4 (2.5)	2.0 (0.05)	1.0 (0.70)	1.9 (0.4)
WF2_D	30.0 (18.7)	5.1 (2.8)	6.6 (3.4)	2.0 (0.01)	1.5 (0.18)	8.6 (0.2)
WF2_S	21.0 (3.0)	3.7 (1.1)	13.6 (3.7)	2.0 (0.01)	1.0 (0.17)	9.2 (0.3)
WF3_D	53.0 (15.7)	6.2 (2.3)	14.5 (1.4)	2.0 (0.02)	1.5 (0.48)	9.1 (0.6)
WF3_S	50.0 (14.7)	7.4 (1.6)	7.0 (3.2)	2.0 (0.02)	1.6 (0.06)	8.6 (0.9)
WF4_D	38.0 (23.6)	1.0 (0.5)	43.1 (14.7)	2.1 (0.07)	1.5 (0.19)	1.0 (0.0)
WF4_S	25.3 (9.1)	0.9 (0.3)	25.8 (3.8)	2.3 (0.17)	0.6 (0.80)	1.0 (0.0)
WF5_D	58.3 (13.6)	4.8 (2.0)	9.4 (4.1)	2.1 (0.00)	1.6 (0.23)	5.0 (2.8)
WF6_D	28.3 (8.0)	8.6 (2.5)	16.8 (2.3)	2.1 (0.01)	2.0 (0.16)	9.2 (0.5)
WF6_S	33.3 (16.7)	7.3 (1.3)	14.3 (4.8)	2.1 (0.01)	1.8 (0.50)	8.0 (0.0)*
WF7_D	55.7 (21.5)	16.8 (6.4)	12.3 (2.2)	2.1 (0.01)	1.9 (0.30)	8.8 (0.1)
WF7_S	61.7 (13.2)	19.1 (10.6)	11.9 (5.0)	2.1 (0.01)	1.9 (0.14)	9.0 (0.5)
WF8_D	51.3 (21.5)	22.6 (14.5)	10.8 (4.1)	2.1 (0.01)	1.8 (0.41)	9.0 (0.4)
WF9_M	36.3 (16.4)	17.1 (10.2)	9.0 (2.4)	2.0 (0.04)	1.6 (0.35)	2.4 (0.2)
WF10_M	34.7 (19.7)	6.6 (0.8)	13.2 (1.8)	2.0 (0.01)	1.8 (0.25)	9.1 (0.3)
WF11_M	46.3 (5.5)	29.3 (19.7)	9.4 (2.3)	2.1 (0.01)	2.0 (0.16)	7.8 (0.8)
WF12_M	27.7 (7.2)	2.8 (1.9)	5.5 (0.3)	2.1 (0.09)	1.1 (0.66)	3.1 (1.4)
WF13_M	34.7 (20.5)	12.1 (6.1)	11.6 (1.6)	2.1 (0.02)	1.6 (0.70)	9.3 (0.7)
WF14_M	52.3 (15.9)	18.9 (15.6)	10.8 (3.6)	2.1 (0.01)	2.0 (0.29)	8.9 (0.1)
**Human samples**						
WF3_D	61.0 (36.5)	3.8 (1.5)	7.0 (2.4)	2.0 (0.02)	1.5 (0.27)	2.6 (0.6)
WF7_D	102.3(35.5)	9.9 (3.0)	14.3 (1.8)	2.1 (0.01)	1.9 (0.08)	8.7 (0.0)*
WF11_M	44.0 (9.2)	4.9 (0.7)	9.6 (1.2)	2.1 (0.01)	1.6 (0.27)	3.2 (0.8)
WF14_M	51.3 (16.2)	4.7 (1.4)	12.9 (2.7)	2.1 (0.01)	1.7 (0.37)	8.2 (0.1)

* The RIN evaluation was made visually, as Agilent Bioanalyzer 2100 Expert software was not able to calculate it during three different measurements.

Table 3S: Reference genes with corresponding qPCR primers.

Gene Symbol	Sequence Accession No	PCR forward (upper) and reverse (lower) primer sequences (5'-3')	Amplicon length (bp)	Location of primer	Targeted splice variants (Transcript ID)
HPRT1	ENSG00000165704	GACTTTGCTTTCCTTGGTCAGG<br>AGTCTGGCTTATATCCAACACTTCG	101	Exon 6<br>Exon 7	ENST00000298556.7<br>ENST00000462974.5<br>ENST00000475720.1
OSBP	ENSG00000110048	AGAGGGCTGGCTCTTCAAAT<br>GGTACCACGGCAGGTATGTC	127	Exon 1<br>Exon 2	ENST00000263847.5
PGK1	ENSG00000102144	CAGTTTGGAGCTCCTGGAAG<br>AGTTGACTTAGGGGCTGTGC	106	Exon 9<br>Exon 10	ENST00000373316.4
SDHA	ENSG00000073578	TGGACCTGGTTGTCTTTGGT<br>CCAGCGTTTGGTTTAATTGG	94	Exon 10<br>Exon 11	ENST00000264932.10<br>ENST00000617470.4<br>ENST00000504309.5<br>ENST00000510361.5

Table 4S: qPCR validation. NO – no additional PCR product from genomic DNA according to GenomeTester software; Cq – crossing point; SD – standard deviation; r^2 – correlation coefficient; LOD – limit of detection.

Gene Symbol	Specificity confirmed by GenomeTester 1.3	Cq (SD) of negative control	Formula for calibration curve*	PCR efficiency calculated from slope (%)	r^2 of calibration curve	Linear dynamic range (cDNA amount in ng)	Cq variantion at LOD as SD	$LOD_{relative\ (ng)}$
HPRT1	NO	37.16 (1.77)	y = -3.2956x + 31.131	101.14	0.99095	0.3125 – 10.0 ng	0.180	0.3125
OSBP	NO	> 40	y = -3.1131x + 31.547	109.52	0.99305	0.3125 – 10.0 ng	0.304	0.3125
PGK1	Additional product from chr X (470 bp)	37.67 (2.4)	y = -3.2892x + 27.071	101.39	0.99957	0.3125 – 10.0 ng	0.078	0.3125
SDHA	NO	36.61 (0.70)	y = -3.4103x + 29.319	96.45	0.99713	0.3125 – 10.0 ng	0.308	0.3125

* Formula for calibration curve: y =ax+b; a=slope, b= Y intercep

Figure 1S. A general study plan. QIAzol - QIAzol Lysis reagent, APTR - Allprotect Tissue Reagent, BME + LB - RTL lysis buffer from RNeasy Fibrous Tissue Mini kit containing beta-mercaptoethanol, Fastprep D/S - Fastprep-24 instrument with lysing matrix D or S tubes, GentleMACS M - GentleMACS Dissociator with M tubes, WF - workflows.

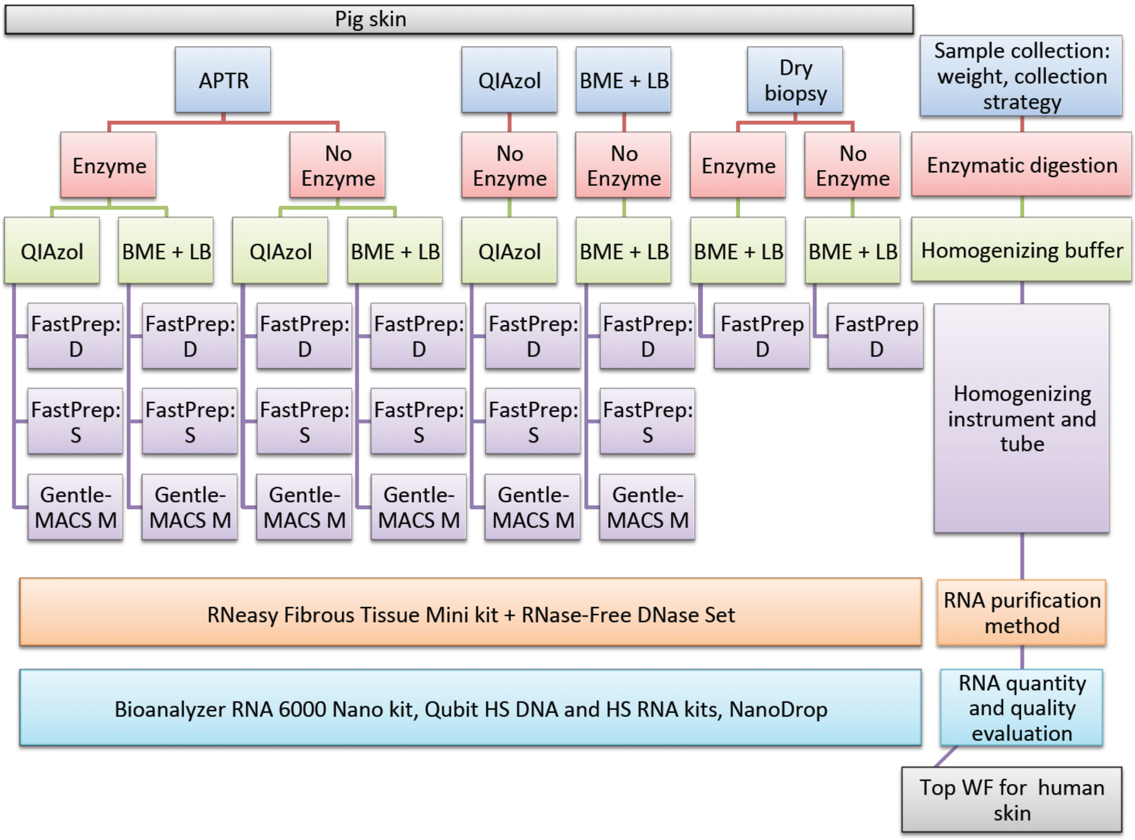

Figure 2S. The Spearman's correlation between weight of biopsy (mg) and total amount of RNA (µg). A – pooled domestic pig skin samples (n = 60), B – pooled human skin samples (n = 12).

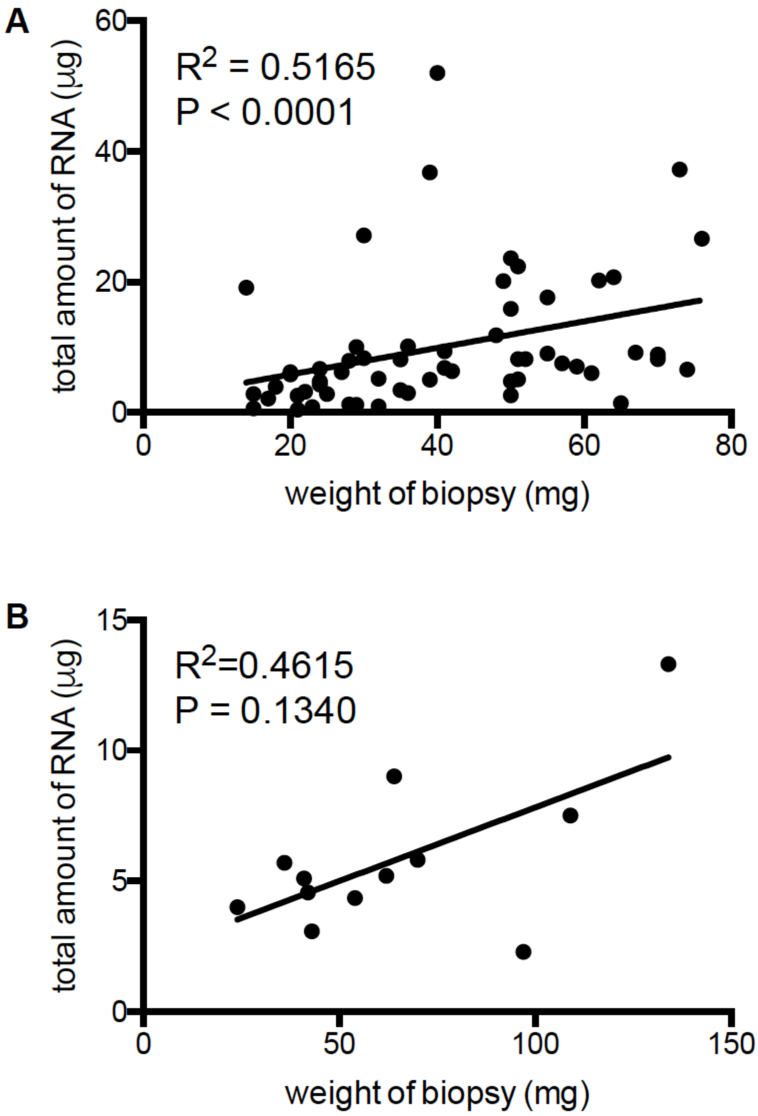

Figure 3S. The domestic pig skin derived RNA quantity and quality values received when using different sample collection/storage strategies. A- total amount of RNA (µg); B – DNA contamination as percentages from whole nucleic acid amount; C – OD 260/280 rate; D – OD 260/230 rate; E – RIN values; Enzymatically digested samples are excluded from subfigures A to E. Results are displayed as mean ± SEM. *P < 0.05, **P < 0.01, ***P < 0.001. F – RNA quality and quantity values received by applying different sample collection strategies ordered according to the RIN values (starting from the group with highest RIN value). The RIN values are represented on top of the columns together with ± SD in brackets. The error bars represent the ± SD. Groups containing term "_Enz" includes also the data from enzymatically digested samples.

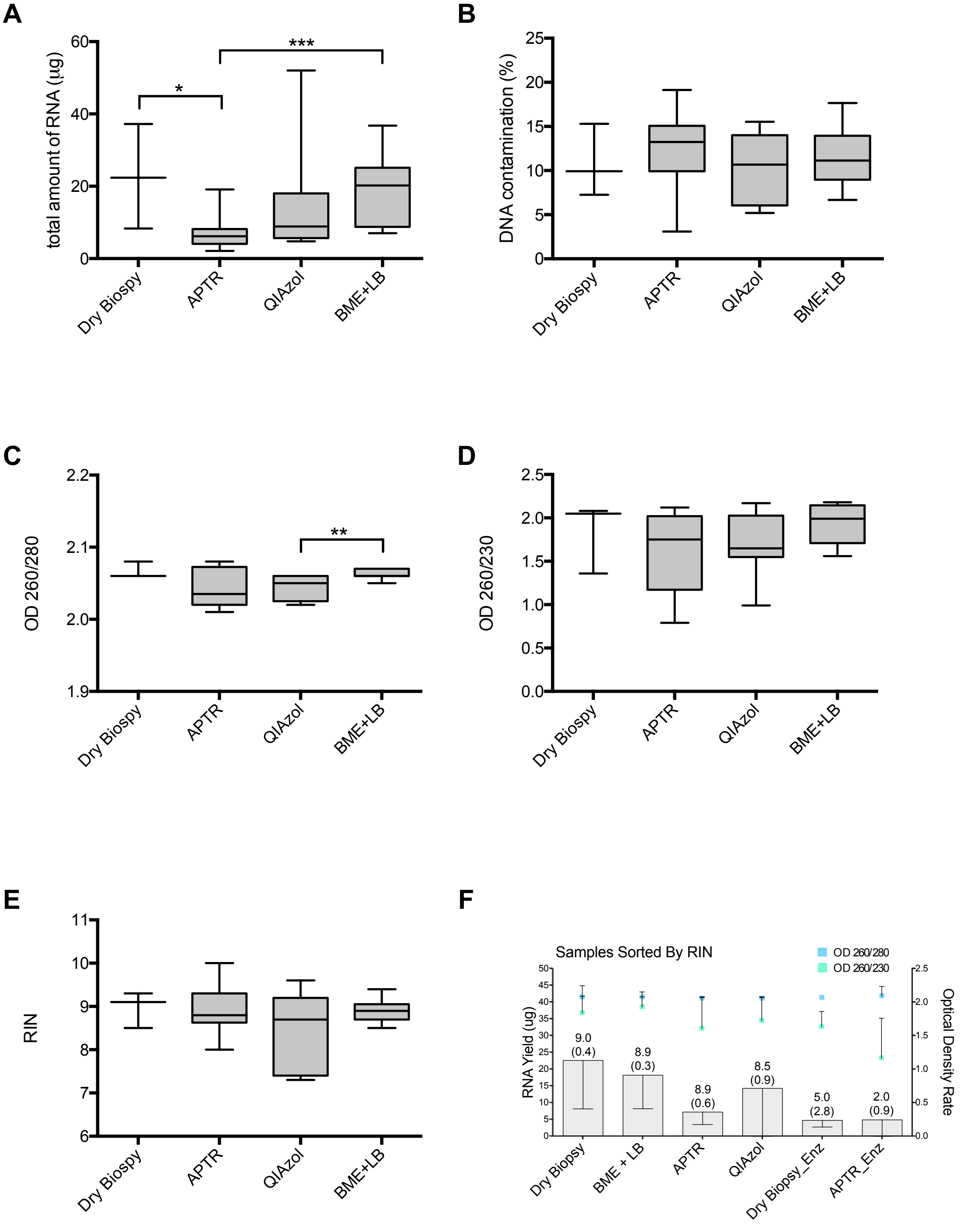

Figure 4S. The domestic pig skin derived RNA quantity and quality values received when using different homogenizing buffers. A- total amount of RNA (μg); B – DNA contamination as percentages from whole nucleic acid amount; C – OD 260/280 rate; D – OD 260/230 rate; E – RIN values; Enzymatically digested samples are excluded from subfigures A to E. Results are displayed as mean ± SEM. *P < 0.05, **P < 0.01, ***P < 0.001. F – RNA quality and quantity values received by applying different homogenizing buffers ordered according to the RIN values (starting from the group with highest RIN value). The RIN values are represented on top of the columns together with ± SD in brackets. The error bars represent the ± SD. Groups containing term "_Enz" includes also the data from enzymatically digested samples.

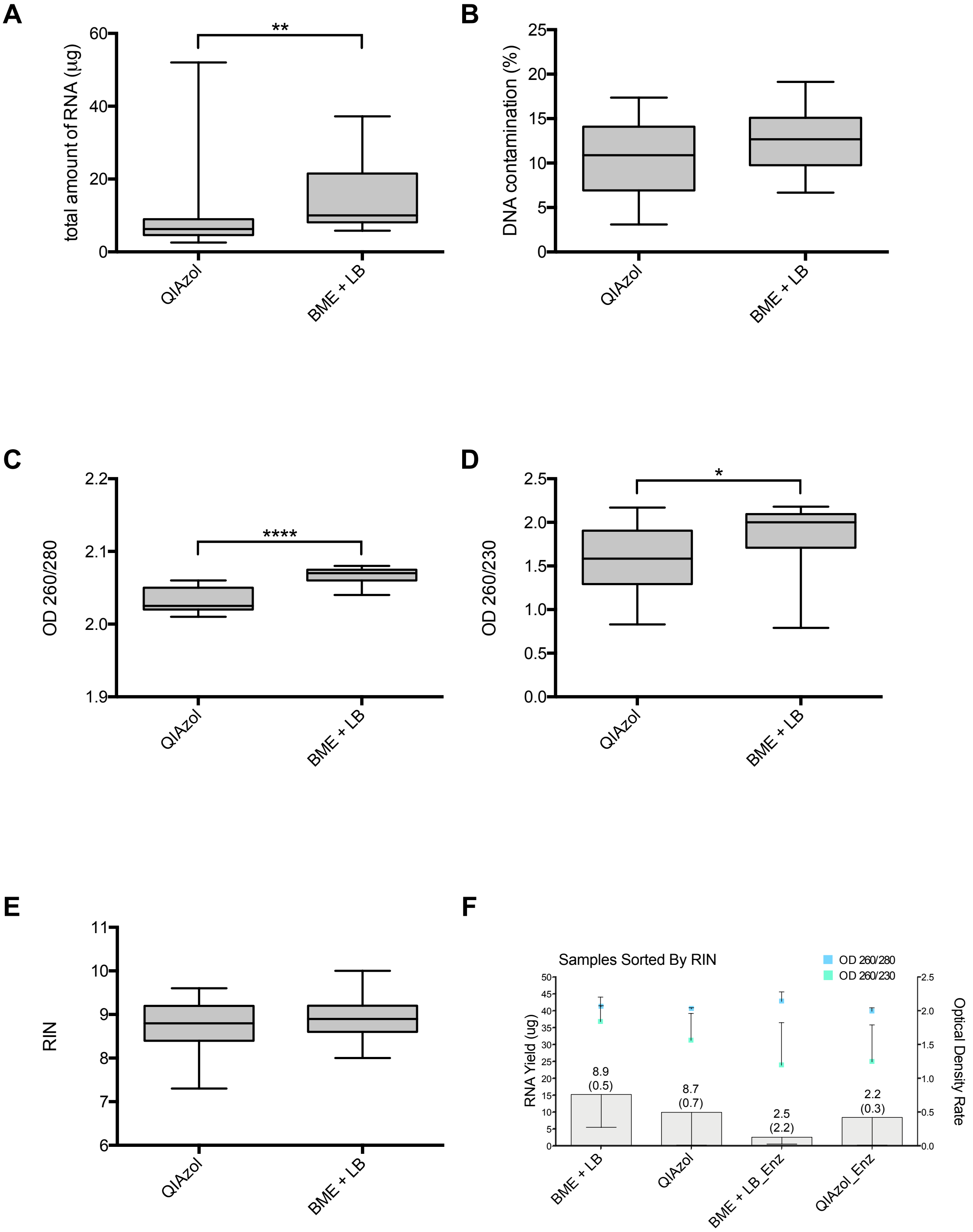

Figure 5S. The domestic pig skin derived RNA quantity and quality values received when using different homogenizing instruments and tubes. A- total amount of RNA (µg); B – DNA contamination as percentages from whole nucleic acid amount; C – OD 260/280 rate; D – OD 260/230 rate; E – RIN values; Enzymatically digested samples are excluded from subfigures A to E. Results are displayed as mean ± SEM. *P < 0.05, **P < 0.01, ***P < 0.001. F – RNA quality and quantity values received by applying different homogenizing instruments and tubes ordered according to the RIN values (starting from the group with highest RIN value). The RIN values are represented on top of the columns together with ± SD in brackets. The error bars represent the ± SD. Groups containing term "_Enz" includes also the data from enzymatically digested samples.

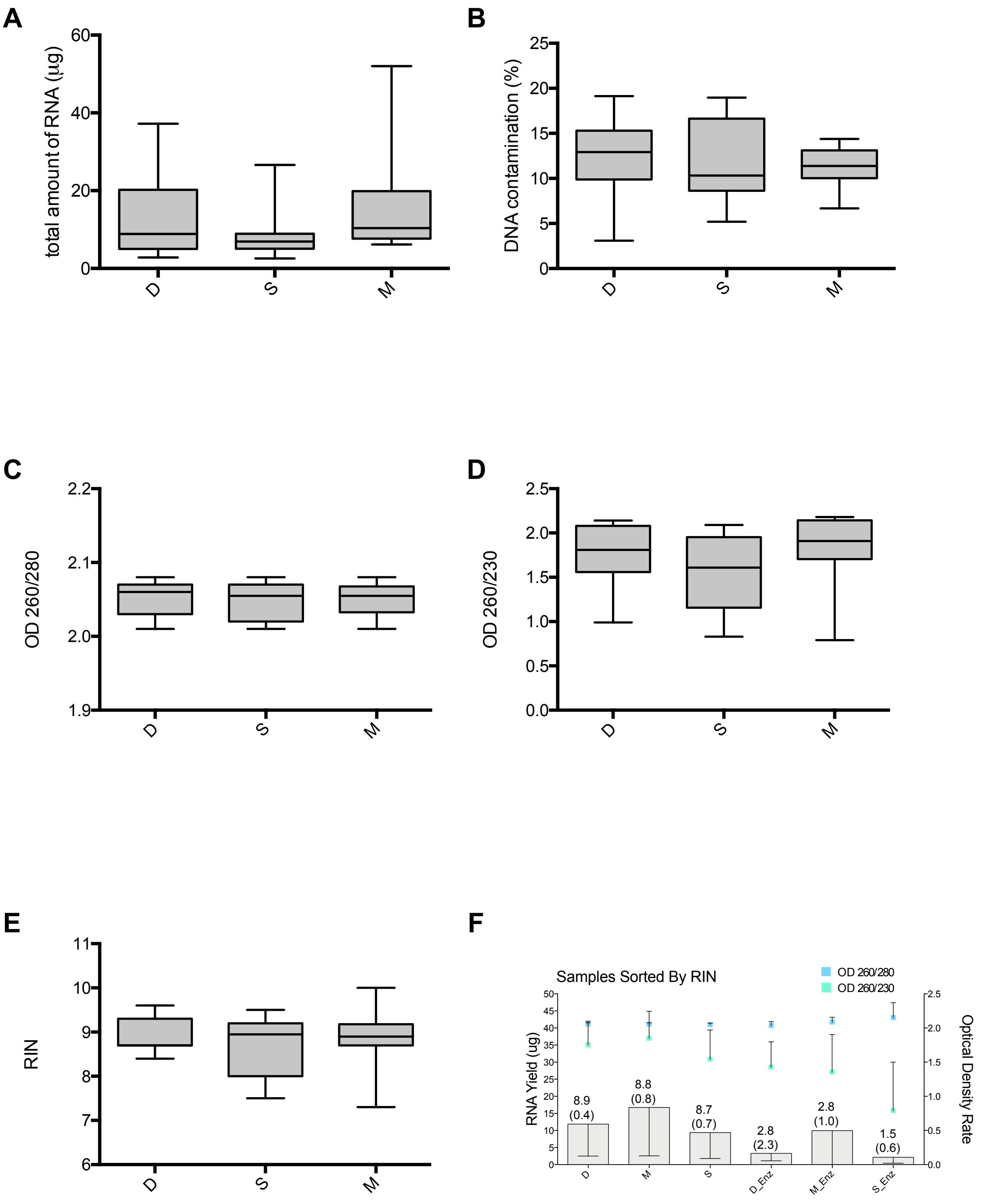

Figure 6S. The human skin derived RNA quantity and quality values received when using different sample collection/homogenizing buffer strategies. A- total amount of RNA (μg); B – DNA contamination as percentages from whole nucleic acid amount; C – OD 260/280 rate; D – OD 260/230 rate; E – RIN values; Results are displayed as mean ± SEM. *P < 0.05, **P < 0.01, ***P < 0.001. F – RNA quality and quantity values received by applying different sample collection/homogenizing buffer strategies ordered according to the RIN values (starting from the group with highest RIN value). The RIN values are represented on top of the columns together with ± SD in brackets. The error bars represent the ± SD.

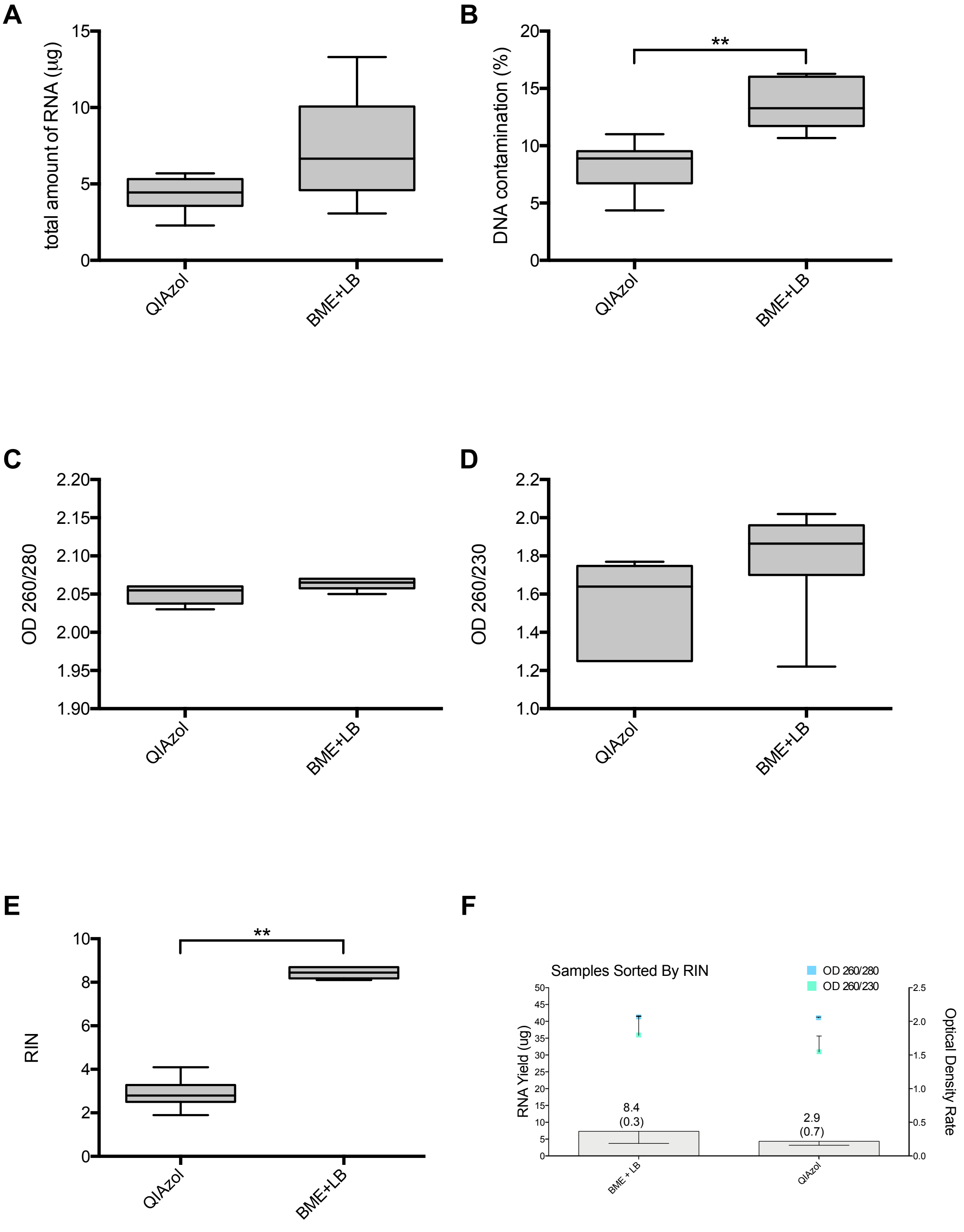

Figure 7S. The human skin derived RNA quantity and quality values received when using different homogenizing instruments and tubes. A- total amount of RNA (μg); B – DNA contamination as percentages from whole nucleic acid amount; C – OD 260/280 rate; D – OD 260/230 rate; E – RIN values; Results are displayed as mean ± SEM. *P < 0.05, **P < 0.01, ***P < 0.001. F – RNA quality and quantity values received by applying different homogenizing instruments and tubes ordered according to the RIN values (starting from the group with highest RIN value). The RIN values are represented on top of the columns together with ± SD in brackets. The error bars represent the ± SD.

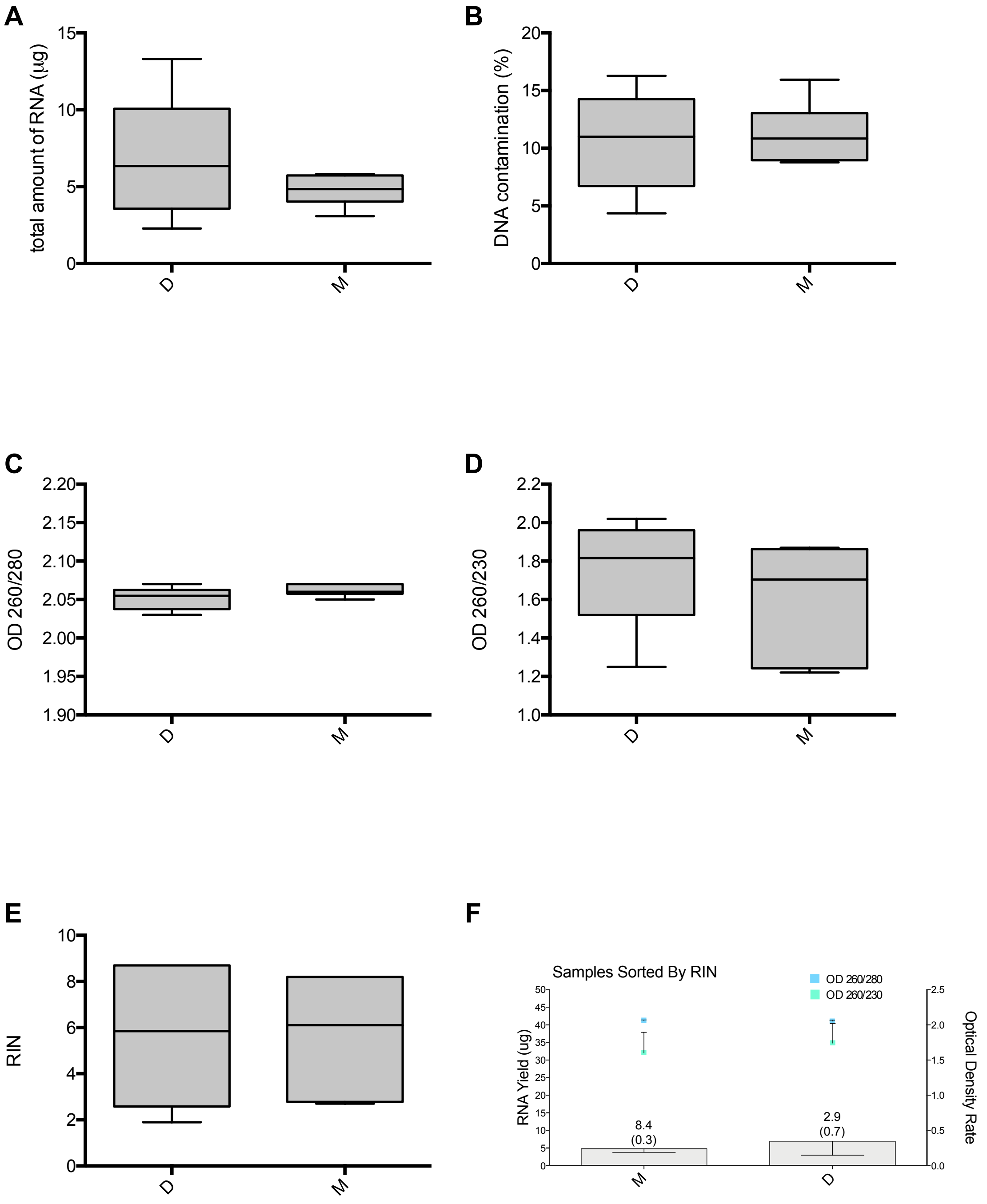

Figure 8S. Human skin derived RNA quality confirmation with gene expression analysis applying qPCR. A-D – correlation analysis between RNA quality (RIN value) and reference gene expression level (ΔCq). E-H – RNA expression level (ΔCq) of different reference genes represented as mean values with standard deviation. For all genes $P > 0.05$.

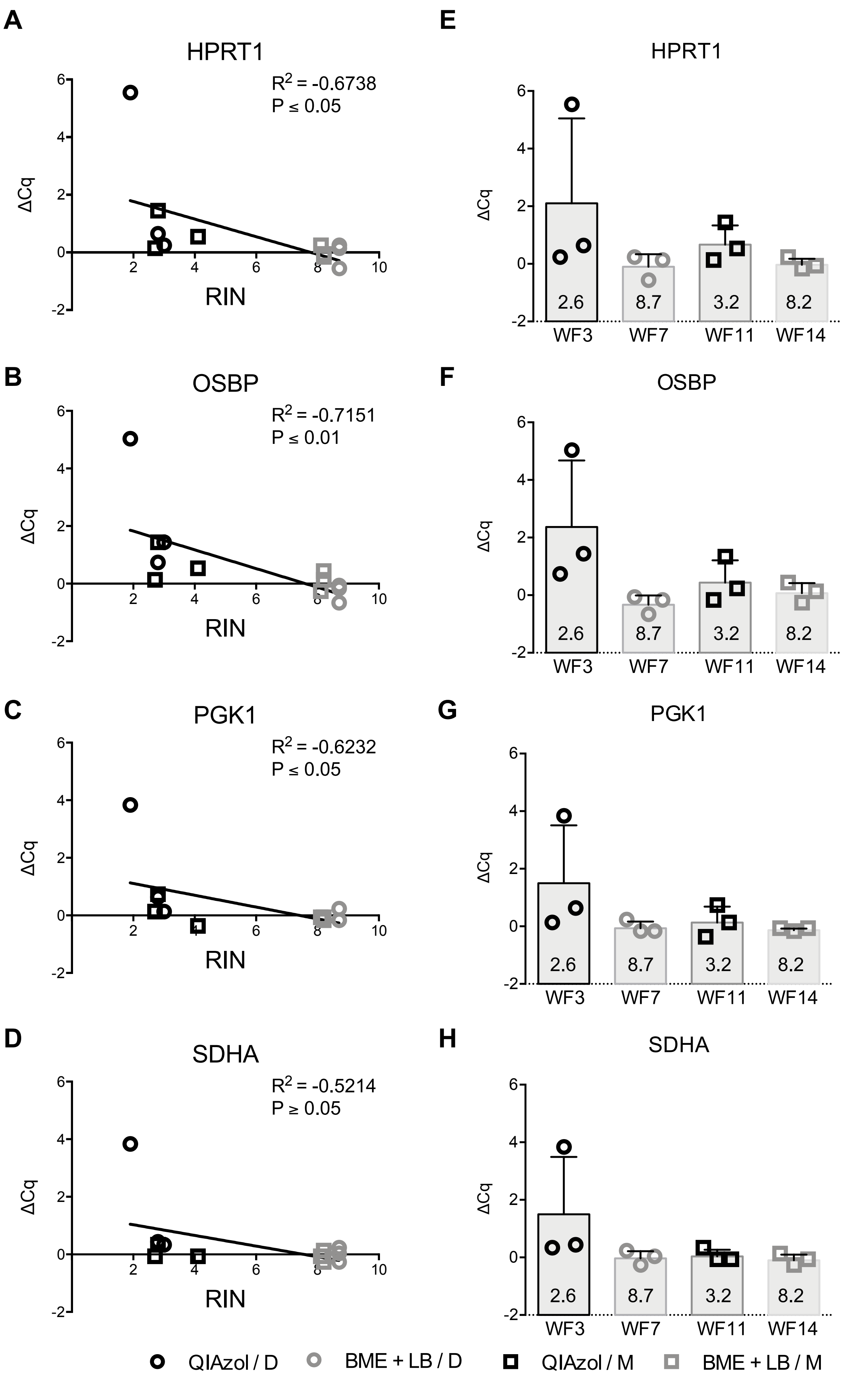

A
HPRT1
R2 = -0.6738
P ≤ 0.05
ΔCq
RIN
B
OSBP
R2 = -0.7151
P ≤ 0.01
C
PGK1
R2 = -0.6232
P ≤ 0.05
D
SDHA
R2 = -0.5214
P ≥ 0.05
E
F
G
H
2.6
8.7
3.2
8.2
WF3
WF7
WF11
WF14
QIAzol / D
BME + LB / D
QIAzol / M
BME + LB / M

Figure 9S: Calibration curves for target and reference genes. Both the formula of calibration curve and correlation coefficient are presented.

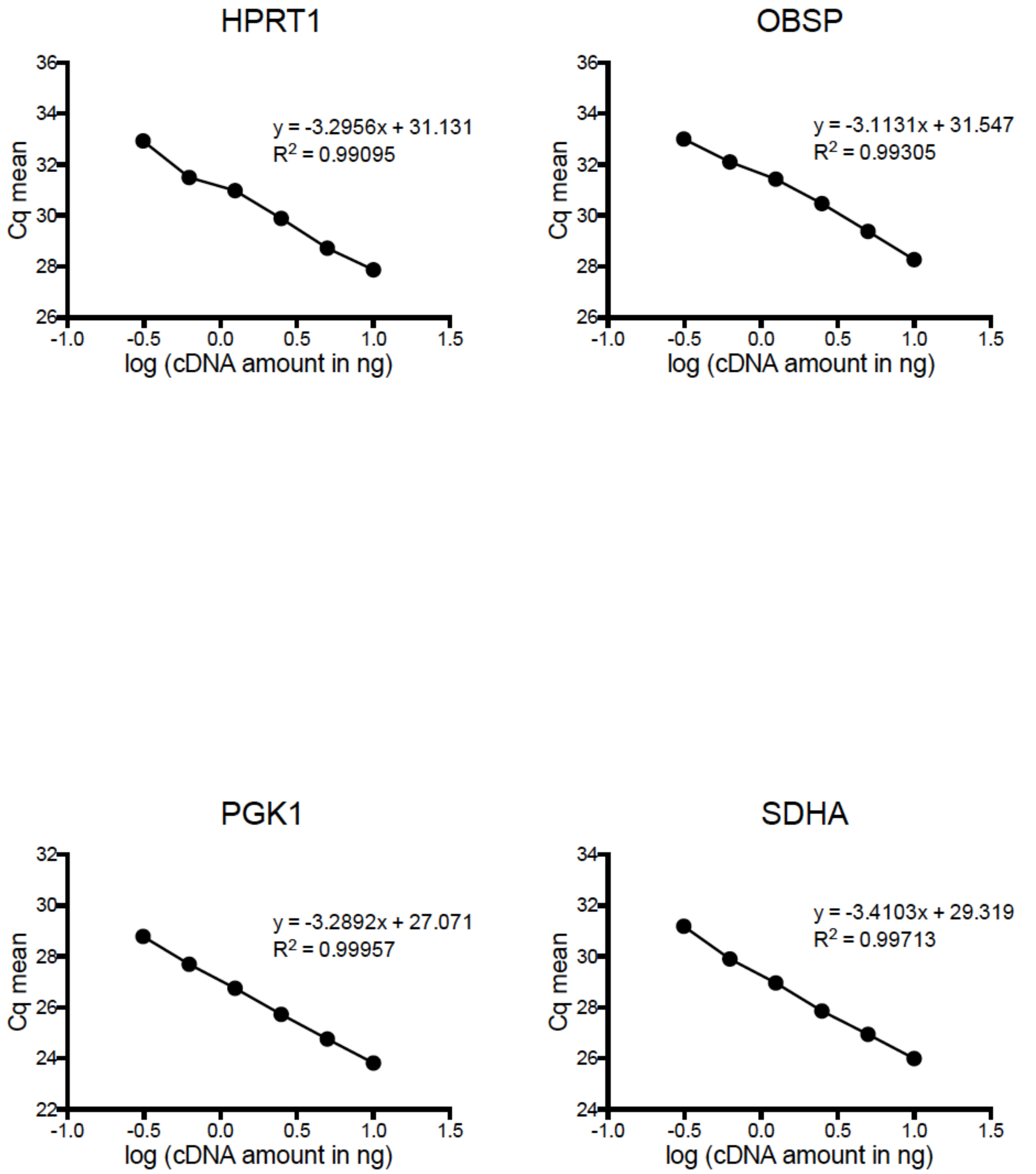

Figure 10S: A – combined The Spearman's correlation between weight of biopsy (mg) and total amount of RNA (µg). Red – pooled domestic pig skin samples (n = 60), Blue – pooled human skin samples (n = 12). B - principal component analysis of RNA quantity (total amount of RNA (µg)) and quality values (OD 280/260, OD 230/260, RIN, DNA contamination %) of domestic pig and human samples.

A

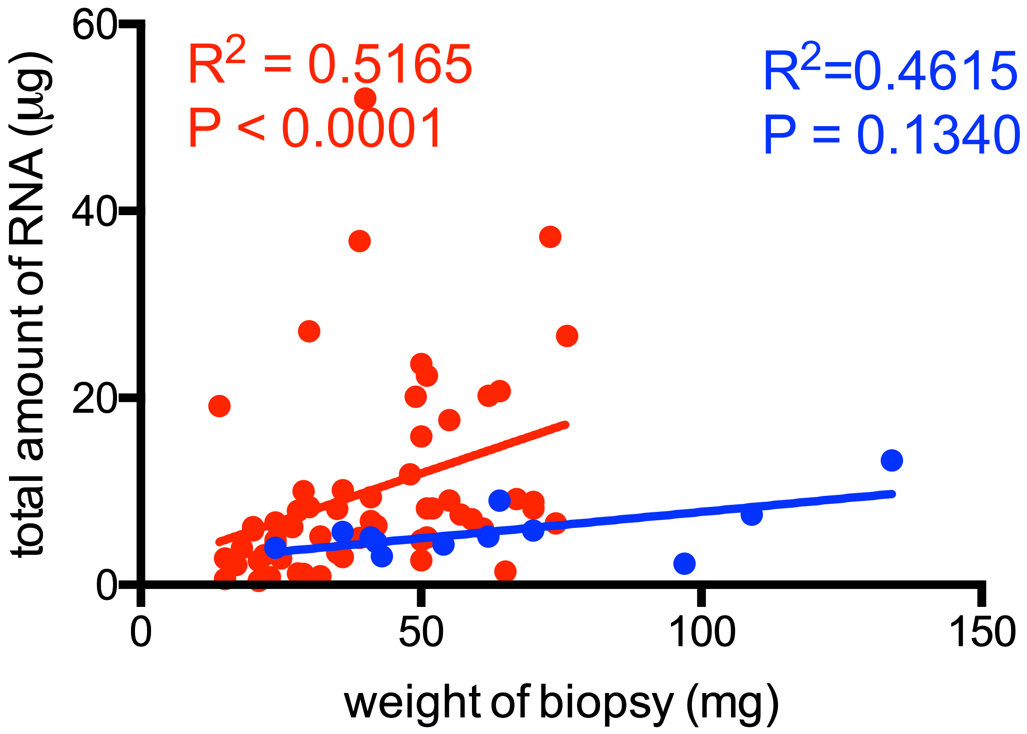

B

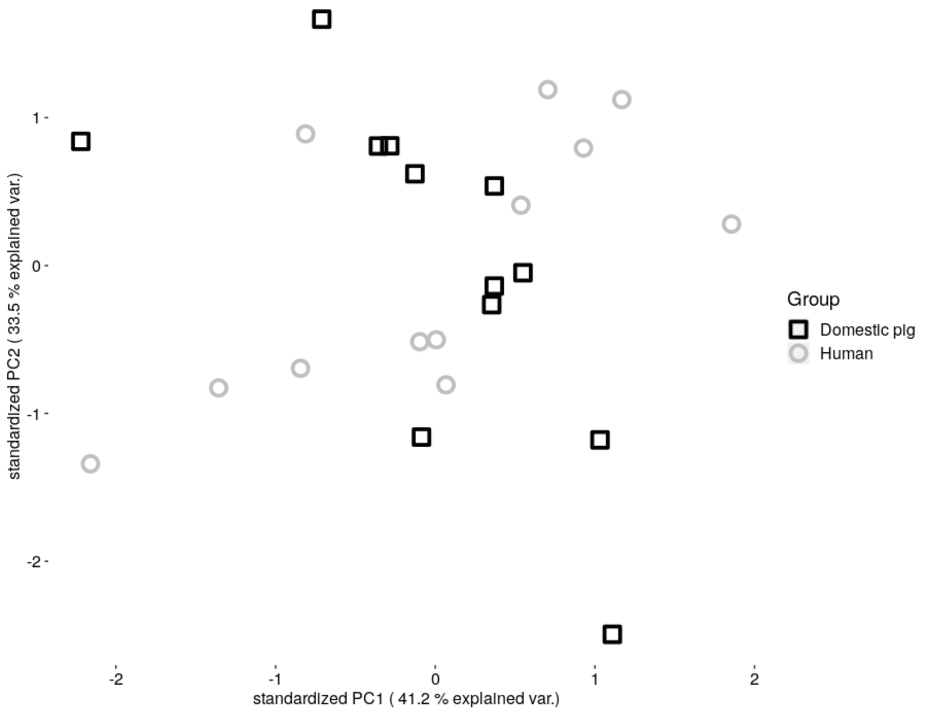

References

1 *User Guide: Qubit dsDNA HS Assay Kits*, MAN0002326 | MP32851, Revision B.0).

2 *User Guide: Qubit RNA HS Assay Kits*, MAN0002327 | MP32852, Revision: A.0).

3 *Agilent 2100 Bioanalyzer 2100 Expert User's Guide*, May 2005).

4 *Agilent RNA 6000 Nano Kit Guide*, 07/2013 G2938-90034 Rev. B).

5 *NanoDrop Nucleic Acid Handbook*, 11/2010).

6 Jaguszewski, M. *et al.* A signature of circulating microRNAs differentiates takotsubo cardiomyopathy from acute myocardial infarction. *Eur Heart J* **35**, 999-1006, doi:10.1093/eurheartj/eht392 (2014).

7 Padhi, B. K., Singh, M., Rosales, M., Pelletier, G. & Cakmak, S. A PCR-based quantitative assay for the evaluation of mRNA integrity in rat samples. *Biomol Detect Quantif* **15**, 18-23, doi:10.1016/j.bdq.2018.02.001 (2018).

8 Hantzsch, M. *et al.* Comparison of whole blood RNA preservation tubes and novel generation RNA extraction kits for analysis of mRNA and MiRNA profiles. *PLoS One* **9**, e113298, doi:10.1371/journal.pone.0113298 (2014).

9 Vartanian, K. *et al.* Gene expression profiling of whole blood: comparison of target preparation methods for accurate and reproducible microarray analysis. *BMC Genomics* **10**, 2, doi:10.1186/1471-2164-10-2 (2009).

10 Malone, J. H. & Oliver, B. Microarrays, deep sequencing and the true measure of the transcriptome. *BMC Biol* **9**, 34, doi:10.1186/1741-7007-9-34 (2011).

11 Wang, Z., Gerstein, M. & Snyder, M. RNA-Seq: a revolutionary tool for transcriptomics. *Nat Rev Genet* **10**, 57-63, doi:10.1038/nrg2484 (2009).

12 *RNeasy Fibrous Tissue Handbook*, 10/2010).

13 Qiagen. *qPCR: RNA Quality and Why It Matters*, 2017).

14 Bar, M., Bar, D. & Lehmann, B. Selection and validation of candidate housekeeping genes for studies of human keratinocytes--review and recommendations. *J. Invest. Dermatol.* **129**, 535-537, doi:10.1038/jid.2008.428 (2009).

15 He, J. Q. *et al.* Selection of housekeeping genes for real-time PCR in atopic human bronchial epithelial cells. *Eur. Respir. J.* **32**, 755-762, doi:10.1183/09031936.00129107 (2008).

16 Turabelidze, A., Guo, S. & DiPietro, L. A. Importance of housekeeping gene selection for accurate reverse transcription-quantitative polymerase chain reaction in a wound healing model. *Wound Repair Regen.* **18**, 460-466, doi:10.1111/j.1524-475X.2010.00611.x (2010).

17 Durrenberger, P. F. *et al.* Selection of novel reference genes for use in the human central nervous system: a BrainNet Europe Study. *Acta Neuropathol* **124**, 893-903, doi:10.1007/s00401-012-1027-z (2012).

18 Untergasser, A. *et al.* Primer3--new capabilities and interfaces. *Nucleic Acids Res* **40**, e115, doi:10.1093/nar/gks596 (2012).

19 Andreson, R., Reppo, E., Kaplinski, L. & Remm, M. GENOMEMASKER package for designing unique genomic PCR primers. *BMC Bioinformatics* **7**, 172, doi:10.1186/1471-2105-7-172 (2006).

20 Bustin, S. A. *et al.* The MIQE guidelines: minimum information for publication of quantitative real-time PCR experiments. *Clin. Chem.* **55**, 611-622, doi:10.1373/clinchem.2008.112797 (2009).

21 Wickham, H. ggplot2: Elegant Graphics for Data Analysis. R package version 3.2.0.
22 R Core Team. R: A language and environment for statistical computing. R version 3.6.0.
